# *Streptococcus gallolyticus* subsp. *gallolyticus* promotes colorectal tumor development

**DOI:** 10.1371/journal.ppat.1006440

**Published:** 2017-07-13

**Authors:** Ritesh Kumar, Jennifer L. Herold, Deborah Schady, Jennifer Davis, Scott Kopetz, Margarita Martinez-Moczygemba, Barbara E. Murray, Fang Han, Yu Li, Evelyn Callaway, Robert S. Chapkin, Wan-Mohaiza Dashwood, Roderick H. Dashwood, Tia Berry, Chris Mackenzie, Yi Xu

**Affiliations:** 1 Center for Infectious and Inflammatory Diseases, Institute of Biosciences and Technology, Texas A&M Health Science Center, Houston, Texas, United States of America; 2 Department of Pathology, Baylor College of Medicine, Houston, Texas, United States of America; 3 Department of Gastrointestinal Medical Oncology, the University of Texas M. D. Anderson Cancer Center, Houston, Texas, United States of America; 4 Epidemiology, the University of Texas M. D. Anderson Cancer Center, Houston, Texas, United States of America; 5 Department of Internal Medicine, University of Texas Health Science Center, Houston, Texas, United States of America; 6 Department of Microbiology and Microbial Genetics, University of Texas Health Science Center, Houston, Texas, United States of America; 7 Harbin Institute of Technology, Harbin, China; 8 Program in Integrative Nutrition and Complex Diseases, Texas A&M University, College Station, Texas, United States of America; 9 Center for Epigenetics and Disease Prevention, Institute of Biosciences and Technology, Texas A&M Health Science Center, Houston, Texas, United States of America; 10 Center for Translational Cancer Research, Institute of Biosciences and Technology, Texas A&M Health Science Center, Houston, Texas, United States of America; 11 Department of Microbial Pathogenesis and Immunology, College of Medicine, Texas A&M Health Science Center, College Station, Texas, United States of America; University of California, San Francisco, UNITED STATES

## Abstract

*Streptococcus gallolyticus* subsp. *gallolyticus* (*Sg*) has long been known to have a strong association with colorectal cancer (CRC). This knowledge has important clinical implications, and yet little is known about the role of *Sg* in the development of CRC. Here we demonstrate that *Sg* promotes human colon cancer cell proliferation in a manner that depends on cell context, bacterial growth phase and direct contact between bacteria and colon cancer cells. In addition, we observed increased level of β-catenin, c-Myc and PCNA in colon cancer cells following incubation with *Sg*. Knockdown or inhibition of β-catenin abolished the effect of *Sg*. Furthermore, mice administered with *Sg* had significantly more tumors, higher tumor burden and dysplasia grade, and increased cell proliferation and β-catenin staining in colonic crypts compared to mice receiving control bacteria. Finally, we showed that *Sg* is present in the majority of CRC patients and is preferentially associated with tumor compared to normal tissues obtained from CRC patients. These results taken together establish for the first time a tumor-promoting role of *Sg* that involves specific bacterial and host factors and have important clinical implications.

## Author summary

Colorectal cancer (CRC) is a leading cause of cancer-related death. The recognition that microbial agents can contribute to the development of CRC raises hope for improving CRC diagnosis and treatment by incorporating both microbial and patient characteristics into clinical strategies. *S*. *gallolyticus* subsp. *gallolyticus* (*Sg*) has been implicated in CRC for decades. Patients with *Sg* infections display a much higher risk of having CRC compared to the general population. Despite this, the precise role of *Sg* in the development of CRC—*i*.*e*., whether this organism plays an active role in the development of tumor or its presence is merely a consequence of the tumor environment being favorable for its colonization of the colon—was unknown. Here using *in vitro* cell cultures and mouse models of CRC, we demonstrate that *Sg* actively promotes colon cancer cell proliferation and tumor growth, suggesting that it is not an innocent “passenger”. These results represent a major step forward in understanding the relationship between *Sg* and CRC. This combined with the prevalence of *Sg* in CRC patients highlight *Sg* as being both clinically relevant and functionally important for CRC.

## Introduction

Colorectal cancer (CRC) is the second to third most common cancer and a leading cause of cancer-related death [1,2]. The human gut is normally exposed to ~ 10^14^ microorganisms, which are increasingly recognized as being intimately involved in the health and disease status of the gut [3]. The identification of specific microbes driving colon tumorigenesis [4–9] raises hope that we may exploit knowledge about specific tumor-promoting microbes to improve cancer diagnosis, prevention and treatment [10–15].

*Streptococcus gallolyticus* subsp. *gallolyticus* (*Sg*) belongs to the *Streptococcus bovis*/*Streptococcus equinus* complex (SBSEC) and was previously known as *S*. *bovis* biotype I [16,17]. This is a Gram-positive, opportunistic pathogen that causes bacteremia and endocarditis in humans. Patients with bacteremia/endocarditis due to *Sg* display a strong association with CRC as shown in numerous case reports and case series [3,12–15,18–29]. A meta-analysis of case reports and case series published between 1970 and 2010 found that patients with *S*. *bovis* infections had ~60% chance of having concomitant colon adenomas/carcinomas [30], much higher than that in the general population. The SBSEC group includes a number of closely related species such as *Sg*, *S*. *pasteurianus*, *S*. *macedonicus* and *S*. *infantarius* [16,31]. Among these different species, *Sg* infection has the strongest association with CRC (~ 7 fold higher risk compared to infections caused by the other species), suggesting the existence of a *Sg*-specific mechanism(s) that promotes the association between the pathogen and CRC. Other similar studies consistently revealed elevated risks of colorectal adenoma/carcinoma for patients with *Sg* infections [14,21–25,32]. These epidemiological studies clearly show a strong association between *Sg* bacteremia/endocarditis and CRC. Due to the overwhelming clinical association, it is recommended that patients with *Sg* infections undergo thorough colon examination. In addition, recent prospective studies followed patients with endocarditis due to *Sg* or other pathogens for several years and found that a significantly higher percentage of *Sg* endocarditis patients developed new colonic neoplasm during the follow-up period, compared to patients with endocarditis caused by other pathogens [33,34]. This suggests that *Sg* plays a role in the early stages of tumor development. On the other hand, CRC patients may be colonized with *Sg* in their colon with no signs of bacteremia or endocarditis [35,36], although the prevalence of *Sg* in CRC patients is not as well defined as the risk for CRC in patients with active *Sg* infections.

Despite a strong clinical association between *Sg* and CRC, the role of *Sg* in CRC development, *i*.*e*. whether it drives colon tumorigenesis or merely colonizes the colon tumor environment, was unknown. In this study, we investigated the effect of *Sg* on colon tumor development using *in vitro* cultured human colon cancer cells and mouse models of CRC. We demonstrated that *Sg* promoted colon cancer cell proliferation in a manner that depends on the specific cell context, bacterial growth phase, and direct contact between bacteria and colon cancer cells. In addition, we showed that *Sg* promoted cell proliferation by up-regulating β-catenin, a central signaling molecule in colon tumorigenesis. Exposure to *Sg* also resulted in larger tumors in a xenograft model. In an azoxymethane (AOM)-induced mouse model of CRC, mice given *Sg* had significantly higher tumor burden, higher average dysplasia grade, increased cell proliferation and β-catenin level in colon crypts compared to control mice. Lastly, we analyzed tumors and adjacent normal tissues from CRC patients. The results indicate that *Sg* is present in the majority of CRC patients and preferentially associates with tumor tissues. These results demonstrate a tumor-promoting role of *Sg* and have important implications with respect to microbial contributions to CRC as well as clinical practices to combat CRC.

## Results

### *Sg* promotes colon cancer cell proliferation

**Fig 1:**
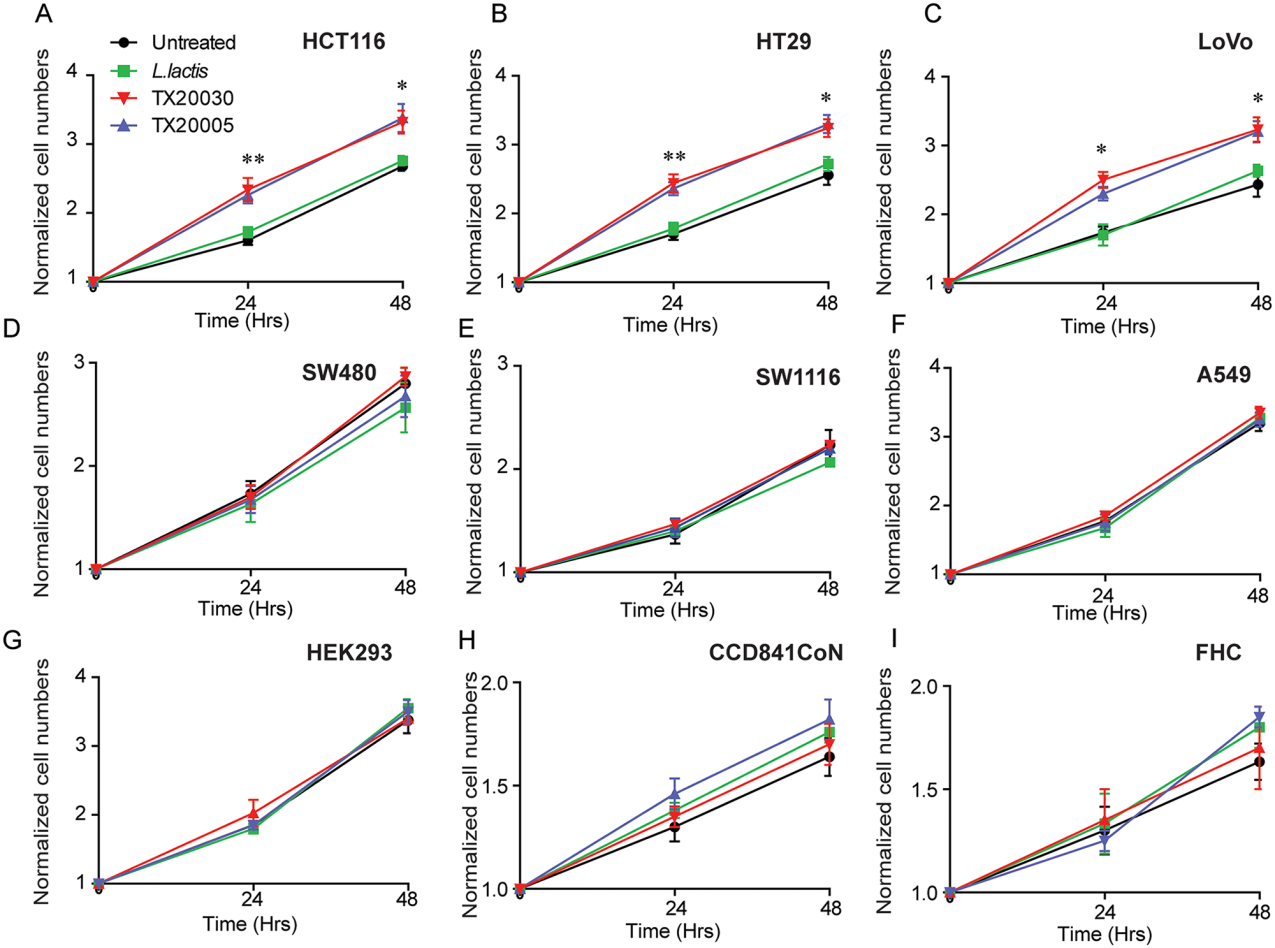
*Sg* stimulates cell proliferation in responsive colon cancer cell lines. Human colon cancer cell lines HCT116 (**A**), HT29 (**B**), LoVo (**C**), SW480 (**D**), and SW1116 (**E**), human lung cancer cell line A549 (**F**), human kidney epithelial cell line HEK293 (**G**), and normal human colon epithelial cell lines CCD841CoN (**H**) and FHC (**I**) were tested. Cells were seeded into the wells of 6-well plates at 1x10^4^ cells per well and incubated for 12 hours. Stationary phase bacteria were washed with sterile phosphate buffered saline, pH 7.4 (PBS) and resuspended in the appropriate cell culture media. Bacterial suspension or media only were then added to the wells at 1x10^2^ cfu/well, and incubated for 24 or 48 hours. Cells were stained with trypan blue and counted in an automated cell counter. Each experiment was done with duplicate wells and repeated at least three times. Data are presented as the mean ± standard error of the mean (SEM). Two-way, two-tailed analysis of variance (ANOVA) followed by Student-Newman-Keuls (SNK) test was used to analyze the data. Significance shown in panels **A**-**C** is for comparison between cells co-cultured with TX20005 and those with *L*. *lactis*. *, *p* < 0.05; **, *p* < 0.01.

The overall effect of *Sg* on cell growth and proliferation was examined using a variety of cell lines. Human colon cancer cell lines HCT116, HT29, LoVo, SW480, SW1116, normal human colon epithelial cell lines FHC and CCD 841 CoN, human kidney epithelial cell HEK293 and human lung cancer cell line A549 were co-cultured with *Sg* strains TX20005 and TX20030, and *Lactococcus lactis* MG1363 (used as a negative control bacterial strain). The number of viable cells was counted after 24 and 48 hours of incubation. We found that, in the presence of the *Sg* strains, HCT116, HT29 and LoVo had significantly more viable cells than colon cancer cells cultured in the presence of *L*. *lactis* or no bacteria (~ 50–60% more at 24 hours and ~ 20–30% more at 48 hours) (Fig 1A–1C). Interestingly, we did not observe any increase in cell numbers for the other cell lines tested including SW480, SW1116, A549, HEK293, CCD841 CoN, and FHC (Fig 1D–1I). These results suggest that *Sg* strains TX20005 and TX20030 promote colon cancer cell growth in a cell context-dependent manner. Therefore, we refer to HT29, HCT116 and LoVo hereafter as “responsive” colon cancer cells, and the others as unresponsive cells.

**Fig 2:**
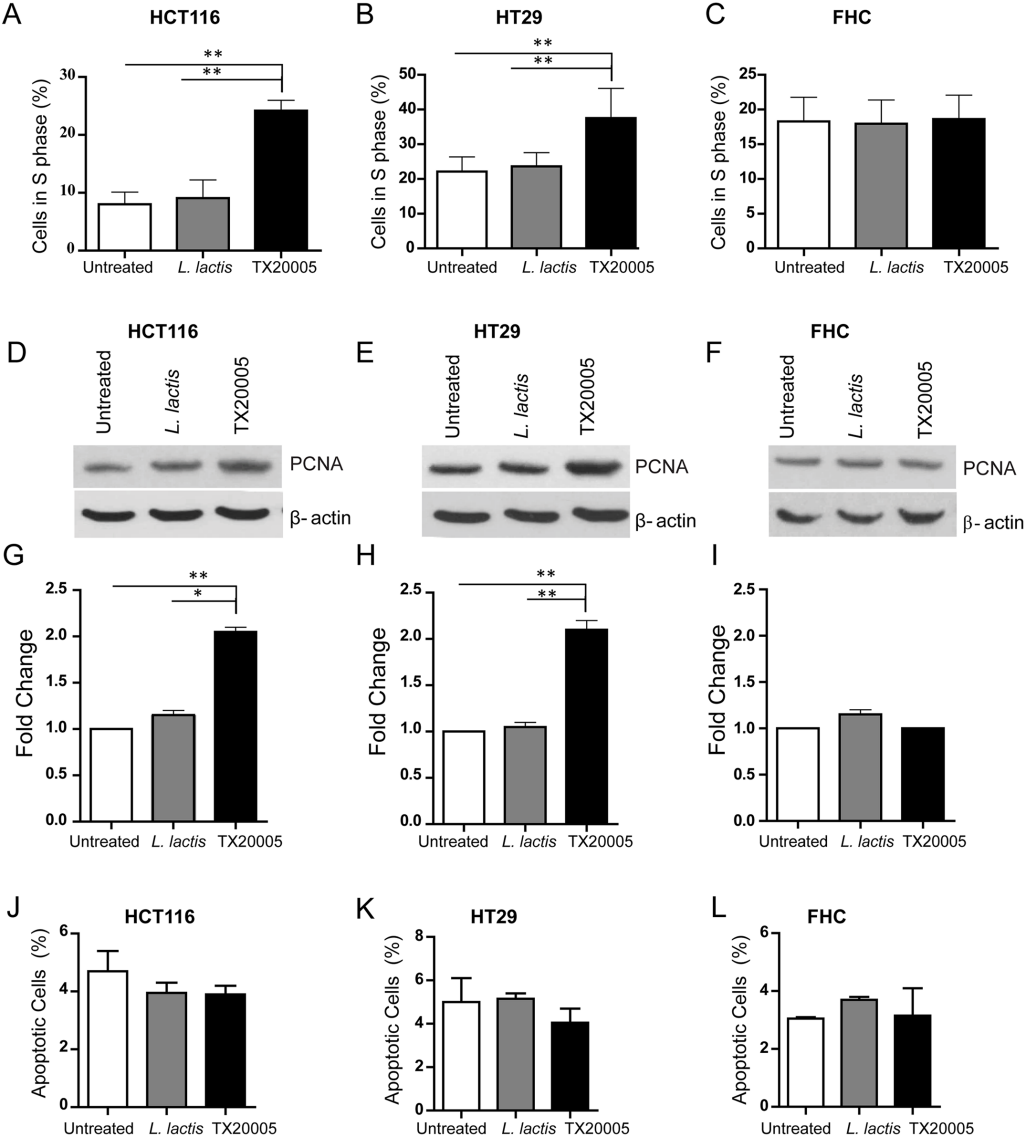
*Sg* promotes cell proliferation but does not affect apoptosis. HCT116, HT29, or FHC cells (~1x10^5^/well) were incubated with *L*. *lactis* or TX20005 (~1x10^5^/well) or media only for 12 hours. Cells were pulsed with 10 μM BrdU for 30 mins, incubated with anti-BrdU antibodies and secondary antibodies, and analyzed by flow cytometry (**A**—**C**). The level of PCNA was determined by western blot assays using total cell lysates from cells co-cultured with TX20005, *L*. *lactis* or media only (**D**—**F**). Representative images are shown. Band intensity was quantified using Image J, normalized to β-actin, and combined from at least three independent experiments (**G**–**I**). Apoptotic cells were detected by staining cells with PI and anti-Annexin V antibodies and secondary antibodies, followed by flow cytometry (**J**–**L**). Each experiment was done with duplicate wells and was repeated at least three times. Data are presented as mean ± SEM. One-way, two tailed ANOVA followed by SNK test was used for statistical analysis. *, *p* < 0.05; **, *p* < 0.01.

The increased number of viable cells after co-culture with *Sg* could be due to increased proliferation, reduced apoptosis, or both. We therefore examined the effect of *Sg* on cell proliferation and apoptosis. Cells co-cultured with *Sg* or *L*. *lactis* were labeled with bromodeoxyuridine (BrdU) and analyzed by flow cytometry. Co-culture with *Sg* TX20005 resulted in ~3 and ~ 1.7-fold increase in the percentage of S phase cells in HCT116 and HT29 cells, respectively, compared to cells incubated with *L*. *lactis* or cells only control (Fig 2A and 2B and S1 Fig). No significant changes in the percentage of S phase cells were observed in FHC cells following incubation with TX20005, as compared to no bacteria or *L*. *lactis*-incubated FHC cells (Fig 2C, S1 Fig). We further determined the level of proliferating cell nuclear antigen (PCNA), a marker for cell proliferation [37], in cells incubated with *Sg*, *L*. *lactis* or cells only. The results showed that HCT116 and HT29 cells incubated with TX20005 had significantly higher levels of PCNA compared to cells co-cultured with *L*. *lactis* or cells only control (Fig 2D, 2E, 2G and 2H). No difference was observed in the PCNA level in FHC cells between the different conditions, as expected (Fig 2F and 2I). These results indicate that *Sg* promotes cell proliferation in responsive cells.

We next examined the effect of *Sg* on cell apoptosis in HCT116, HT29, and FHC cells co-cultured with TX20005, *L*. *lactis*, or media only. The cells were stained with anti-Annexin V antibodies and propidium iodide followed by flow cytometry analysis. No significant difference was observed in the percentage of apoptotic cells between the different groups in any of the cell lines (Fig 2J–2L and S2 Fig). To further confirm this, we compared the level of cleaved caspase 3 and observed no difference between the different conditions in any of the cell lines tested (S3 Fig). Taken together, these results indicate that *Sg* does not affect cell apoptosis, but promotes colon cancer cell proliferation in a cell context-dependent manner.

### The proliferation-promoting effect of *Sg* is *Sg*-specific and depends on bacterial growth phase and direct contact between bacteria and responsive cells

**Fig 3:**
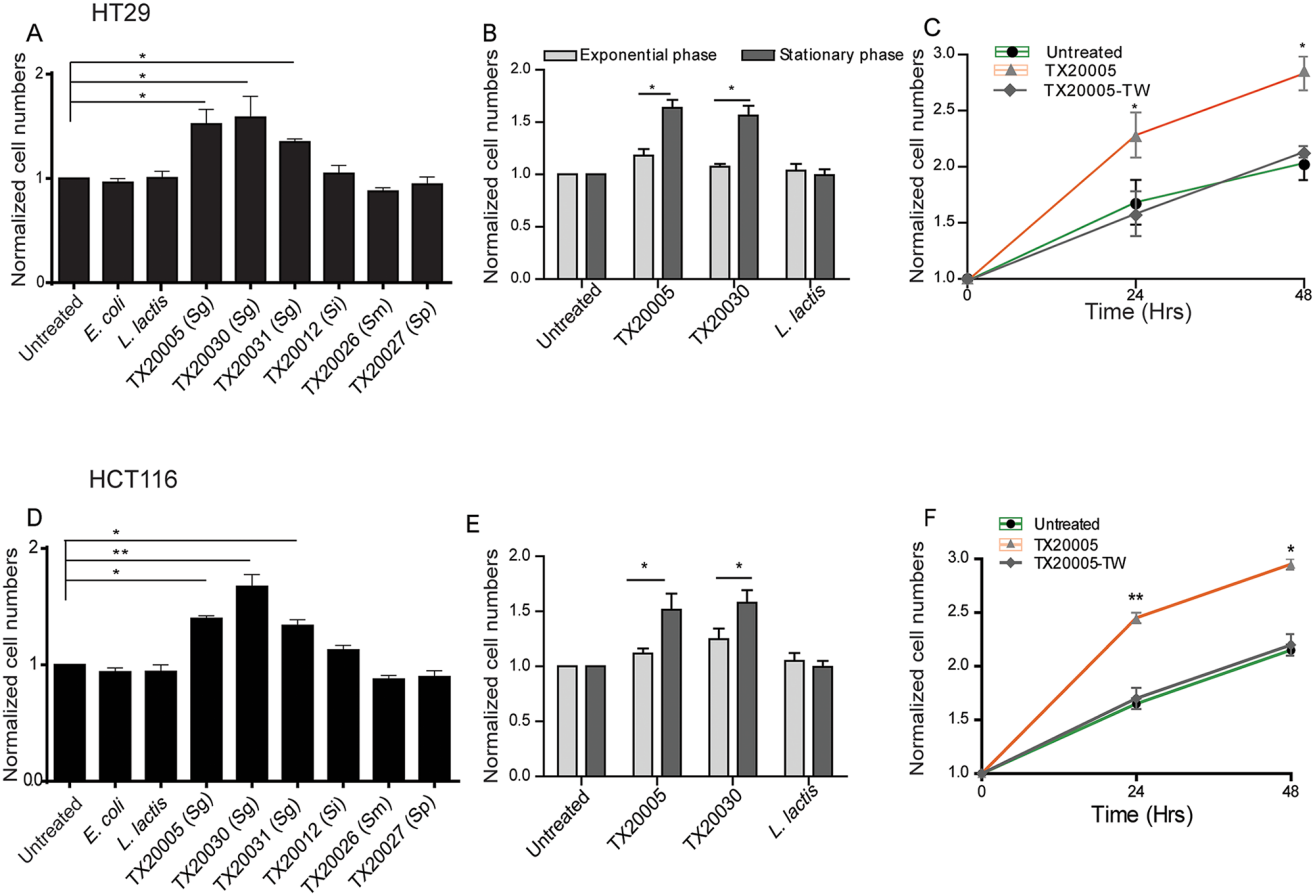
Promotion of cell proliferation requires *Sg*-specific factors and depends on bacterial growth phase and direct contact with CRC cells. **A** and **D**. Species closely related to *Sg* do not promote cell proliferation. Stationary phase bacteria were added to HT29 (**A**) and HCT116 (**D**) cells, co-cultured for 24 hours and viable cell numbers enumerated. Sg, *S*. *gallolyticus* subsp. *gallolyticus*; Si, *Streptococcus infantarius*; Sm, *S*. *gallolyticus* subsp. *macedonicus*; Sp, *S*. *gallolyticus* subsp. *pasteurianus*. **B** and **E**. Promotion of cell proliferation requires stationary but not exponential phase *Sg*. TX20005 bacteria harvested at exponential or stationary phase of growth were added to HT29 (**B**) and HCT116 (**E**) cells and co-cultured for 24 hours. Viable cell numbers were enumerated. **C** and **F**. Promotion of cell proliferation requires direct contact between *Sg* and responsive cells. Stationary phase TX20005 bacteria were added to transwell inserts (0.4 μm pore) (TX20005-TW) or directly to cells and co-cultured with HT29 (**C**) and HCT116 (**F**) cells for 24 and 48 hours. Viable cell numbers were enumerated. Data are presented as the mean ± SEM. Each experiment was done with duplicate wells and was repeated at least three times. Data in panels **A** and **D** were analyzed using one-way, two-tailed ANOVA, followed by SNK test. Data in panels **B** and **E** were analyzed by unpaired, two-tailed t test, stationary phase vs. exponential phase. Data in panels **C** and **F** were analyzed using two-way, two-tailed ANOVA followed by SNK test. Significance shown in **C** and **F** is for comparison between cells co-cultured with TX2005 directly and cells cultured with TX20005-TW. *, *p* < 0.05; **, *p* < 0.01.

We next examined the effect of an expanded panel of bacterial strains on HT29 and HCT116. The panel included *Sg* strains TX20005, TX20030 and TX20031, and strains of closely related species within the SBSEC*—S*. *infantarius* (TX20012), *S*. *macedonicus* (TX20026), and *S*. *pasteurianus* (TX20027). *E*. *coli* strain XL-1 Blue and *L*. *lactis* were included as negative control bacteria. All three *Sg* strains significantly increased HT29 and HCT116 cell numbers at 24 (Fig 3A and 3D) and 48 hour (S4A and S4B Fig) time points, whereas none of the other bacterial strains had any effect.

Bacterial growth under co-culture conditions were determined (S5 Fig). There was no significant difference in the growth curve between the different strains as analyzed by ANOVA. TX20026 showed virtually the same growth curve as TX20005. For TX20012, TX20027 and *L*. *lactis*, the bacterial counts were lower than TX20005 at 6 hours. However, there was no significant difference in the bacterial counts between these strains and TX20005 after 6 hours.

In the co-culture experiments described above, the bacteria added to the wells were from stationary phase cultures. We examined the effect of exponential phase cultures of TX20005, TX20030 and *L*. *lactis* on HT29 and HCT116 cells. In contrast to stationary phase bacteria, exponential phase TX20005 or TX20030 did not cause any significant increase in HT29 (Fig 3B) or HCT116 (Fig 3E) cell numbers at 24 hours compared to the no bacteria controls. Thus, the results suggest that the ability of *Sg* to promote cell proliferation is growth phase-dependent.

We next examined whether secreted bacterial factors or bacterial metabolites in the culture supernatant were sufficient to promote colon cancer cell growth. Supernatants from stationary phase cultures of TX20005, TX20030 and *E*. *coli* were collected and filtered to remove any residual bacteria. HT29 and HCT116 cells were cultured in media only or media supplemented with the culture supernatants. Bacterial culture supernatants were insufficient to promote cell proliferation in HT29 and HCT116 cells (S6A and S6B Fig). The inability of *Sg* culture supernatants to stimulate host cell proliferation could be due to the possibility that the proliferation-promoting effect of *Sg* required bacteria to directly contact host cells. It could also be due to that the factors/metabolites in the culture supernatants were unstable and required a continuous presence of live bacteria in the culture. To distinguish these two possibilities, we used a transwell system in which bacteria were cultured in inserts with permeable membranes of 0.4 μm pore size. This pore size allows the passage of secreted bacterial factors and metabolites but not bacteria. Culturing *Sg* bacteria in transwells resulted in a complete loss of the proliferation-promoting effect of TX20005 on both HT29 and HCT116 cells (Fig 3C and 3F). HT29 cells were also incubated with either heat-killed bacteria or bacterial lysates, prepared from stationary phase culture. We did not observe any increase in the cell number compared to the control group under either treatment conditions (S7 Fig), suggesting that live bacteria are necessary to produce the observed effect on proliferation shown in Fig 1.

Taken together, these results suggest that the proliferation-promoting effect of *Sg* is dependent on *Sg*-specific factors, bacterial growth phase and direct contact between live bacteria and responsive cells.

### Adherence of *Sg* to both responsive and unresponsive cells

Since direct contact between bacteria and cancer cells is required to promote cell proliferation, we investigated the ability of *Sg* to adhere to responsive and unresponsive cell lines. The results showed that both TX20005 and TX20030 adhered to HCT116, HT29, and A549 cells at a similar level (~ 20% of the initial inoculum) and slightly higher to SW1116 and SW480 (~ 30% of the initial inoculum). Adherence to CCD 841 CoN colonic epithelial cells was significantly lower than to the colon cancer cell lines (~15% of the initial inoculum) (S8A Fig). The ability of *Sg* strains to invade these cells was also investigated using gentamicin protection assay. Hardly any intracellular bacteria were detected, suggesting that the two *Sg* strains are poorly or non-invasive (S8B Fig). We further investigated the adherence ability of stationary and exponential phase TX20005 and TX20030. Exponential phase TX20005 and TX20030 adhered to HCT116 cells significantly less than stationary phase bacteria (S8C Fig).

Together these results suggest that the ability of *Sg* to adhere to responsive cells is also growth phase-dependent, consistent with the growth phase-dependency observed in cell proliferation assays. On the other hand, *Sg* strains are able to adhere to unresponsive cells as well as to responsive cells, suggesting there may be multiple interactions between *Sg* and different cell lines.

### *Sg* promotes cell proliferation in a β-catenin dependent manner

**Fig 4:**
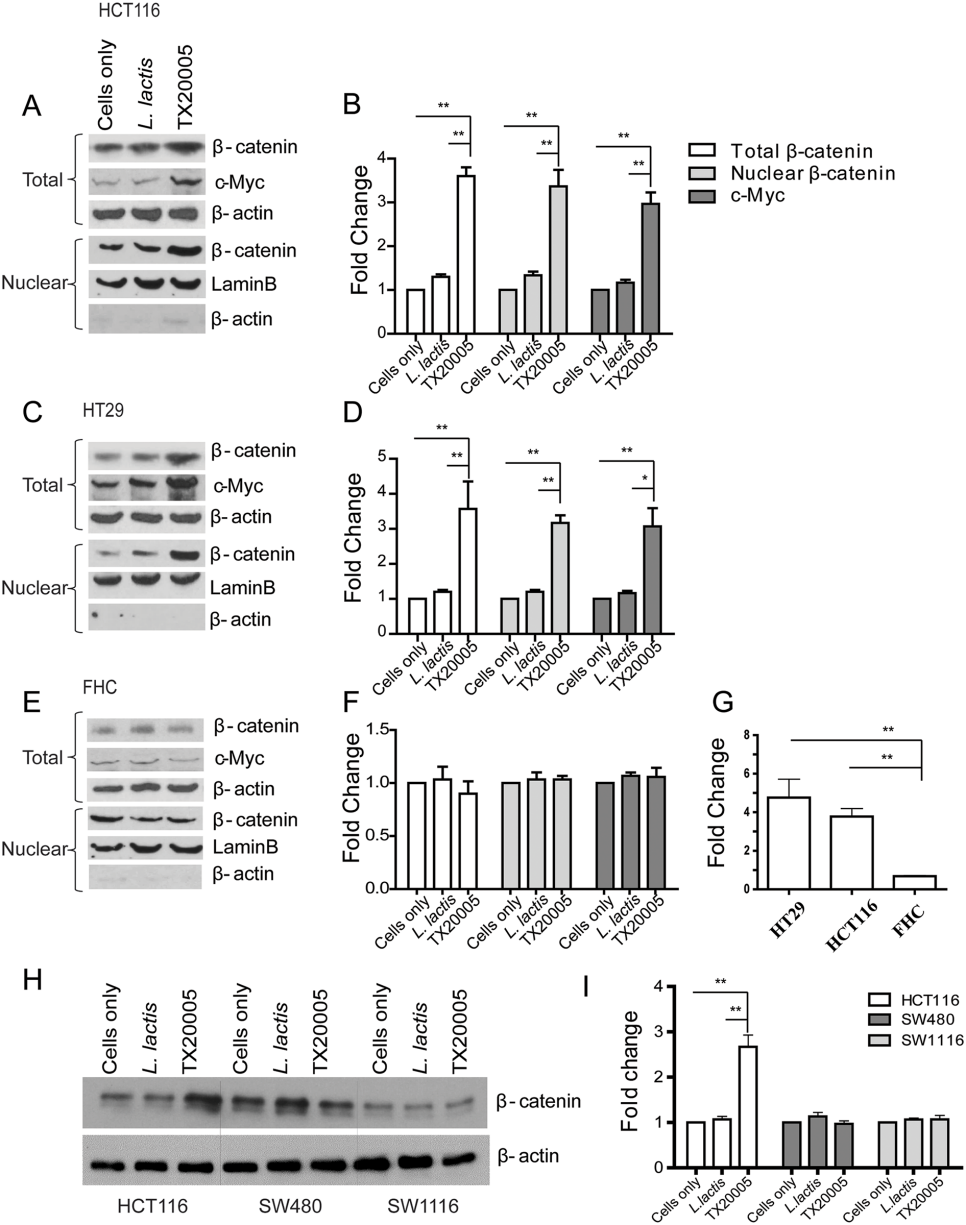
*Sg* increases the level of β-catenin, c-Myc and cyclin D1 in HCT116 and HT29 cells. Approximately 1x10^5^ cells/well were incubated with bacteria (~10^5^ cfu/well) or media only for 12 hours in a 6 well plate. Whole cell or nuclear lysates as well as RNA were extracted and analyzed by western blot assays using specific antibodies (**A**-**F**, **H**, and **I**) or RT-qPCR (**G**). Representative images are shown (**A**, **C**, **E** and **H**). **A** and **B**, HCT116; **C** and **D**, HT29; **E** and **F**, FHC. Band intensity was quantified using Image J, normalized to β-actin or lamin B first and then normalized to the cells only control. **G**. *Sg* increased the expression of cyclin D1 in HCT116 and HT29 cells. ΔΔCT was first normalized to GAPDH then to cells cultured in media only. Results in panels **B**, **D**, **F**, **G** and **I** were combined from at least 3 experiments. Data are presented as mean ± SEM. Data were analyzed using one-way, two-tailed ANOVA followed by SNK test. *, *p* < 0.05; **, *p* < 0.01.

The Wnt/β-catenin signaling pathway regulates cell fate and proliferation and is a critical pathway in colon tumorigenesis [38–40]. We investigated the effect of *Sg* on β-catenin in responsive and unresponsive cells. For HCT116 and HT29 cells, co-culture with TX20005 led to a significantly elevated level of total β-catenin compared to cells co-cultured with *L*. *lactis* or no bacteria after 12 hours of incubation (Fig 4A–4D). In contrast, no increase in β-catenin level was observed in unresponsive FHC, SW480 and SW1116 cells following *Sg* co-culture (Fig 4E, 4F, 4H and 4I). In addition, *Sg* had no effect on the level of β-catenin in A549 cells (S9 Fig). Upon activation, β-catenin is translocated into the nuclei and triggers the enhanced expression of downstream targets, such as c-Myc and cyclin D1 [41]. We examined the level of nuclear β-catenin. The results showed that HCT116 and HT29 cells co-cultured with TX20005 had significantly increased nuclear β-catenin compared to cells co-cultured with *L*. *lactis* or cells only (Fig 4A–4D). No change in nuclear β-catenin was observed in FHC cells under the same experimental conditions (Fig 4E and 4F). In accordance with this observation, the level of c-Myc (Fig 4A–4D) and cyclin D1 (Fig 4G) in HCT116 and HT29 was also significantly increased following co-culture with TX20005 compared to that in the control groups (Fig 4A–4D). No increase in the level of c-Myc (Fig 4E and 4F) or cyclin D1 (Fig 4G) was observed in FHC cells, as expected. In A549 cells, we also observed no changes in the level of c-Myc (S9 Fig). Taken together, these results suggest that incubation of responsive cells with *Sg* results in up-regulation of β-catenin and its oncogenic downstream targets.

**Fig 5:**
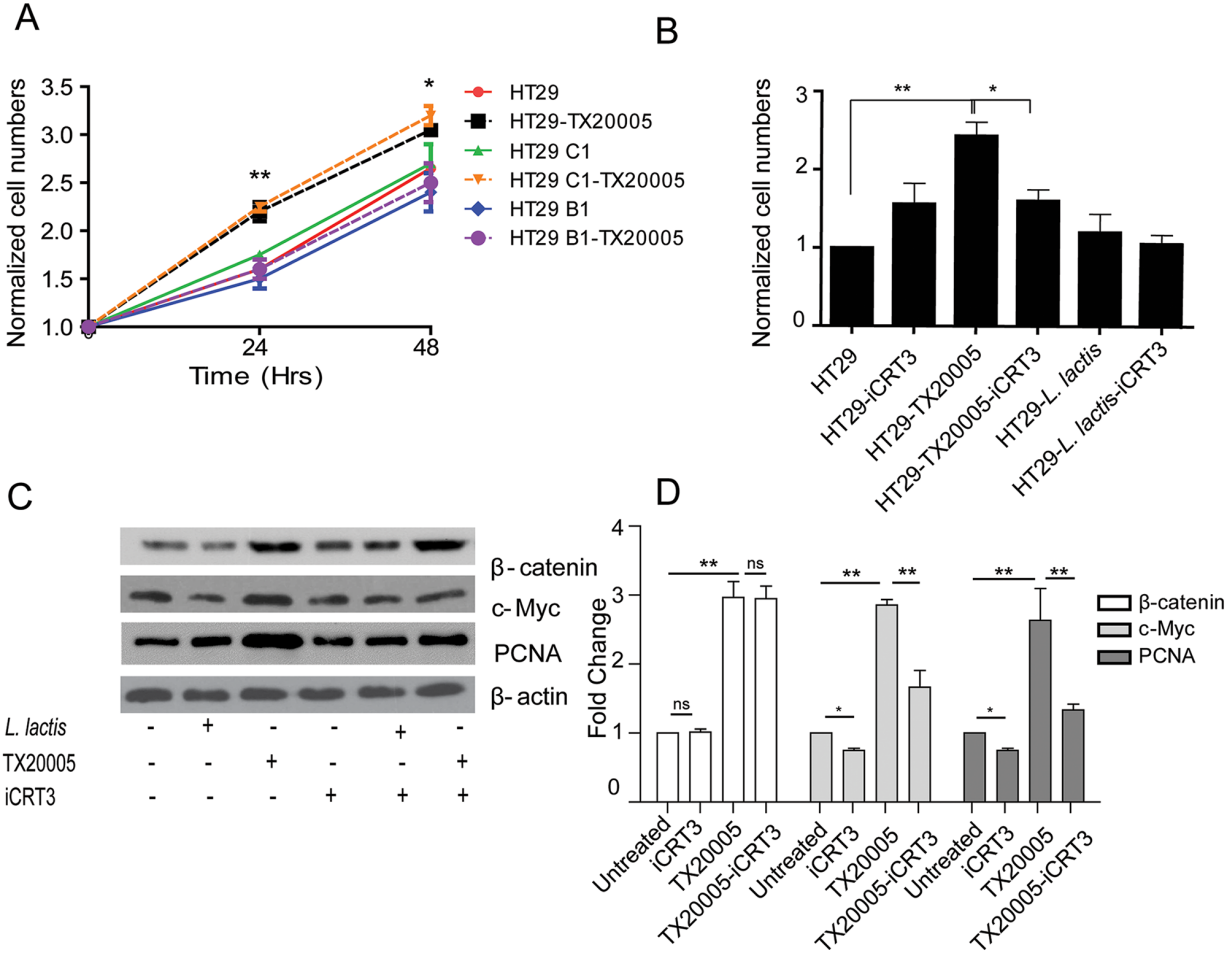
*Sg* promotes cell proliferation in a β-catenin dependent manner. **A**. Knockdown of β-catenin abolished the effect of *Sg*. Cell proliferation assays were performed as described in the legend for Fig 1. Untransfected HT29 cells, β-catenin stable knockdown HT29 cells (HT29-B1) or HT29 cells transfected with a control shRNA (HT29-C1) were used. Data was analyzed using two-way two-tailed ANOVA followed by SNK test. Significance shown panel **A** is for comparison between HT29 cells co-cultured with TX20005 and HT29-B1 with TX20005. **B**—**D**. Inhibition of β-catenin transcriptional activity by iCRT3 rendered cells unresponsive to *Sg*. **B**. Stationary phase TX20005 or *L*. *lactis* bacteria were added to the wells (~1x10^2^ cfu/well) in the presence or absence of iCRT3 (25 μM), incubated for 24 hours and viable cells enumerated. **C** and **D**. Stationary phase TX20005 or *L*. *lactis* were added to cells (~10^5^ cfu/well) as described in the legend for Fig 4 and incubated for 12 hours in the presence or absence of iCRT3. Total cell lysates were prepared and subject to western blot assays to compare β-catenin, c-Myc and PCNA protein levels. Representative images are shown (**C**). Band intensity was quantified using Image J, normalized to β-actin first and then to the cells only control (**D**). Data are presented as the mean ± SEM. Each experiment was done with duplicate wells and was repeated at least three times. Data in panels **B** and **D** were analyzed by using unpaired, two-tailed t test. *, *p* < 0.05; **, *p* < 0.01.

To determine the role of β-catenin in *Sg*-mediated cell proliferation, β-catenin stable knockdown cells were generated using two specific shRNA. Knockdown was confirmed using western blot assays (S10A Fig). In co-culture experiments, β-catenin knockdown completely abolished the effect of *Sg* on cell proliferation, whereas cells transfected with control shRNA showed a similar increase in cell numbers as untransfected cells (Fig 5A and S10B Fig). To further confirm this, we used a β-catenin responsive transcription (CRT) inhibitor iCRT3, which disrupts β-catenin-TCF4 interaction [42]. In the presence of iCRT3, TX20005 co-culture with HT29 cells did not increase cell proliferation compared to the control groups (Fig 5B). We next examined the effect of TX20005 on the level of c-Myc and PCNA in the presence of iCRT3. Treatment of HT29 cells with iCRT3 significantly reduced the effect of TX20005 on c-Myc and PCNA expression (Fig 5C and 5D). To determine if β-catenin knockdown or iCRT3 treatment affected bacterial adherence to HT29 cells, we performed adherence assays. We did not observe any significant change in the adherence of TX20005 to either β-catenin knockdown cells or cells treated with iCRT3 compared to untransfected or untreated cells (S10C Fig). Taken together, these results indicate that promotion of cell proliferation by *Sg* is through up-regulation of β-catenin dependent signaling.

### *Sg* promotes tumor growth in a xenograft model

**Fig 6:**
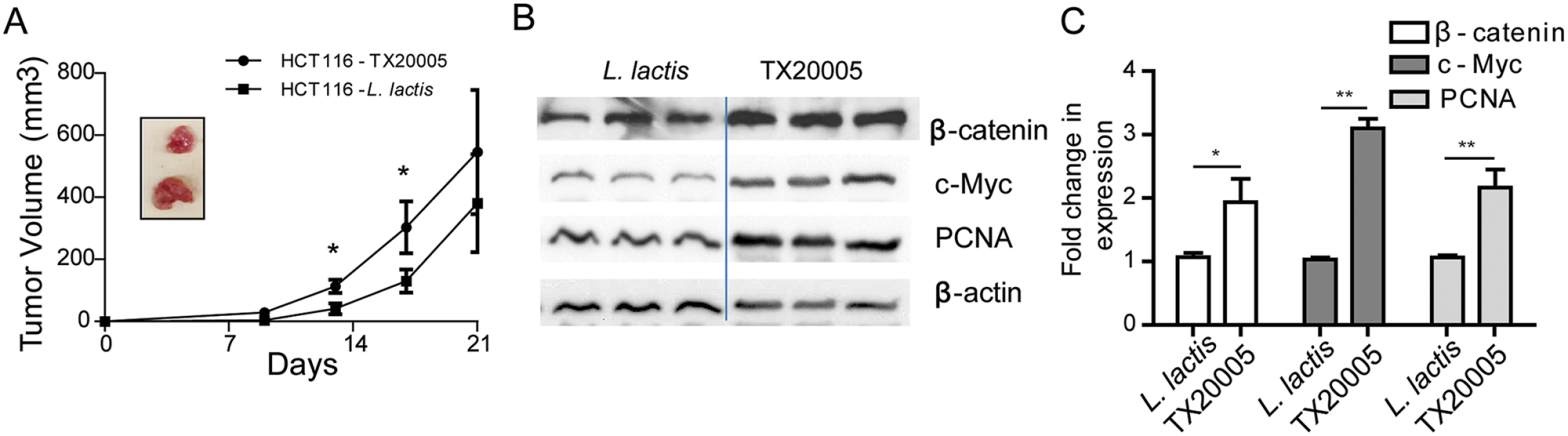
*Sg* treatment promotes tumor growth in a xenograft model. **A**. *Sg*-treated cells developed larger tumors in nude mice. ~ 1x 10^6^ HCT116 cells were treated with TX20005 or *L*. *lactis*, mixed with Matrigel and injected into the dorsal flap of nude mice (n = 5/group) as described in the Methods and Materials section. Tumor size was measured at the indicated time point with a digital caliper. **B**-**C**. *Sg*-treated xenografts had higher levels of β-catenin, c-Myc and PCNA compared to *L*. *lactis*-treated ones. Tumors were collected on day 21. Three tumors were randomly selected from each group. Protein extracts were analyzed by western blot assays (**B**). Protein level was normalized to β-actin first and then to *L*. *lactis*-treated controls (**C**). Data is presented as the mean ± SEM. Statistical analysis was done using unpaired, two-tailed t test. *, *p* < 0.05; **, *p* < 0.01. Significance shown in panel A is of comparison between TX20005-treated and *L*. *lactis*-treated HCT116 cells at day 13 and 19, respectively.

HCT116 cells cultured with TX20005 or *L*. *lactis* were injected into nude mice and tumor growth monitored (Fig 6A). Starting from day 13, TX20005-treated cells formed significantly larger tumors than *L*. *lactis*-treated cells. Expression of β-catenin, c-Myc and PCNA was analyzed in tumors obtained at day 21 (Fig 6B and 6C). A significant increase in the levels of β-catenin, c-Myc and PCNA were observed in tumors from TX20005-treated cells compared to those from *L*. *lactis*-treated cells. We also tested the effect of *Sg* using the non-responsive SW480 cells. The results showed that TX20005-treated SW480 cells did not form bigger tumors compared to cells treated with *L*. *lactis* (S11 Fig). These results indicate that TX20005 treatment promoted tumor growth in the xenograft model, and that this growth promotion requires responsive cells.

### *Sg* promotes colon tumor development in an AOM-induced mouse model of CRC

**Fig 7:**
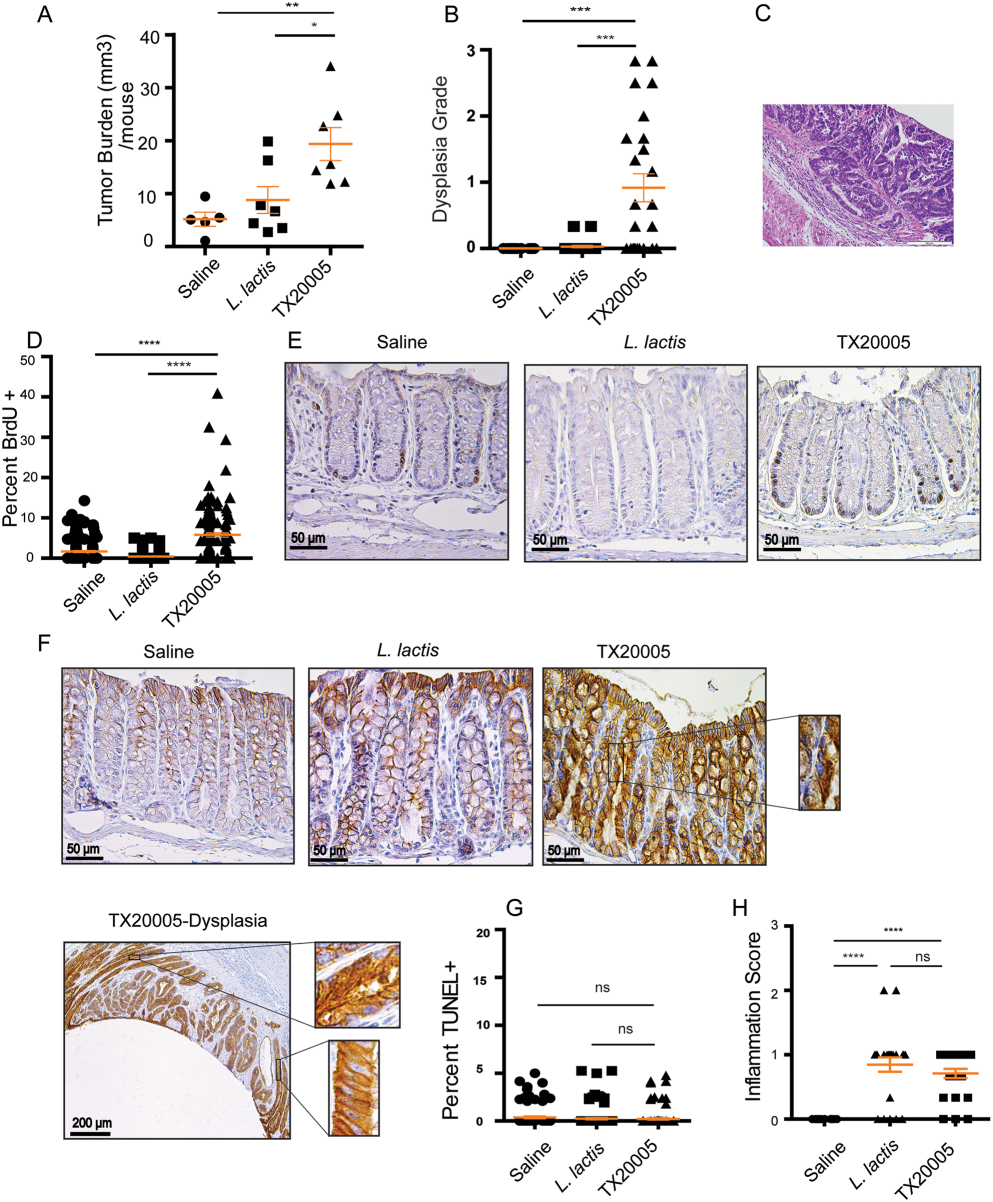
*Sg* promotes colon tumor development in an AOM-induced mouse model of CRC. **A-H**. This was performed as described in the Methods and Materials section. Briefly, A/J mice were administered 2 weekly i.p. injections of AOM, followed by treatment with Amp for 1 week and then oral gavage of bacteria or saline for 24 weeks. Colons were visually examined for macroscopic tumors. Tumor burden (**A**) was calculated. H&E stained colon sections were evaluated for dysplasia (**B**). An image from the TX20005-treated group with a dysplasia grade of 3.5 is shown (**C**). Proliferating cells were determined by staining colon sections for BrdU incorporation (**D** and **E**). Colon sections were also stained for β-catenin (**F**). Apoptosis was assessed by performing TUNEL assays (**G**). H&E stained colon sections were also evaluated for inflammation and average inflammation score for each treatment group is shown (**H**). n = 5 for saline, n = 7 for *L*. *lactis* and TX20005, respectively. All colon evaluation was done by blinded observers. Data is presented as mean ± SEM. Statistical analysis was performed using one-way, two-tailed ANOVA followed by SNK test. *, *p* < 0.05; **, *p* < 0.01; ***, *p* < 0.001; ****, *p* < 0.0001.

To further evaluate the role of *Sg* in the development of colon tumor, we used an AOM-induced mouse model of CRC. This model is commonly used to represent sporadic CRC. Mice were treated with 2 doses of AOM followed by antibiotic treatment for a week and then orally gavaged with TX20005, *L*. *lactis* or saline for 24 weeks. Colons were harvested for visual examination for macroscopic tumors. Overall, most of the tumors were found in the distal portion of the colon. We observed that *Sg*-treated mice had higher tumor burden compared to both the saline and *L*. *lactis* control groups (Fig 7A).

H&E stained colon sections were evaluated. Colons from *Sg*-treated mice showed a significantly higher average dysplasia grade compared to those from *L*. *lactis*-treated or saline control mice (Fig 7B). Adenocarcinomas were observed in *Sg*-treated mice but not in the control groups (Fig 7C). We next examined cell proliferation and apoptosis in mouse colonic crypt cells. *Sg*-treated mice had a significantly higher percentage of proliferating cells (BrdU^+^) in the colonic crypts compared to *L*. *lactis-* or saline-treated control groups (Fig 7D and 7E). In addition, *Sg*-treated mice had higher levels of β-catenin in the colon epithelium as compared to *L*. *lactis*-treated or saline controls (Fig 7F). In contrast, we did not observe any significant difference in the percentage of apoptotic cells between the different treatment groups as determined by TUNEL assays (Fig 7G, S12 Fig). These results are consistent with the observations from our *in vitro* cell culture assays.

Inflammation in the colon of mice was also scored. Both *Sg*- and *L*. *lactis*-treated groups displayed significantly higher average inflammation scores compared to the saline group. However, overall inflammation in these groups was mild and there was no apparent difference between the *Sg*- and *L*. *lactis*-treated groups (Fig 7H). We further measured the level of cytokines TNFα, IL-1β, IL-6, IL-17, IL23 and Cox-2 in tumor and normal tissues collected from the mouse colon using RT-qPCR. There was no significant difference between TX20005 and *L*. *lactis* treated groups in any of the cytokines tested (S13 Fig). These results suggest that *Sg* and *L*. *lactis* trigger similar inflammatory responses.

We further tested the effect of *Sg* on tumor development using a different shorter procedure, in which mice were treated with four doses of AOM and gavaged with bacteria for 12 weeks. Similar to the results from the first longer procedure, a significant increase in tumor numbers was observed in *Sg*-treated mice compared to the saline control (S14A Fig). When compared to the *L*. *lactis* group, mice gavaged with TX20005 also had more tumors; however, the difference was not statistically significant (*p* = 0.08). Tumor burden also displayed a similar trend as that observed in the longer procedure, in which *Sg*-treated mice had a higher average tumor burden than the other two groups (S14B Fig). In the shorter procedure, however, the difference was not statistically significant (*p* = 0.06 vs. *L*. *lactis*-treated mice) perhaps due to reduced duration, less bacterial gavage or more AOM injections in this second procedure. Overall, results from the two procedures show a consistent trend pointing towards *Sg* acting as a promotional agent for tumor development in the mouse colon.

**Fig 8:**
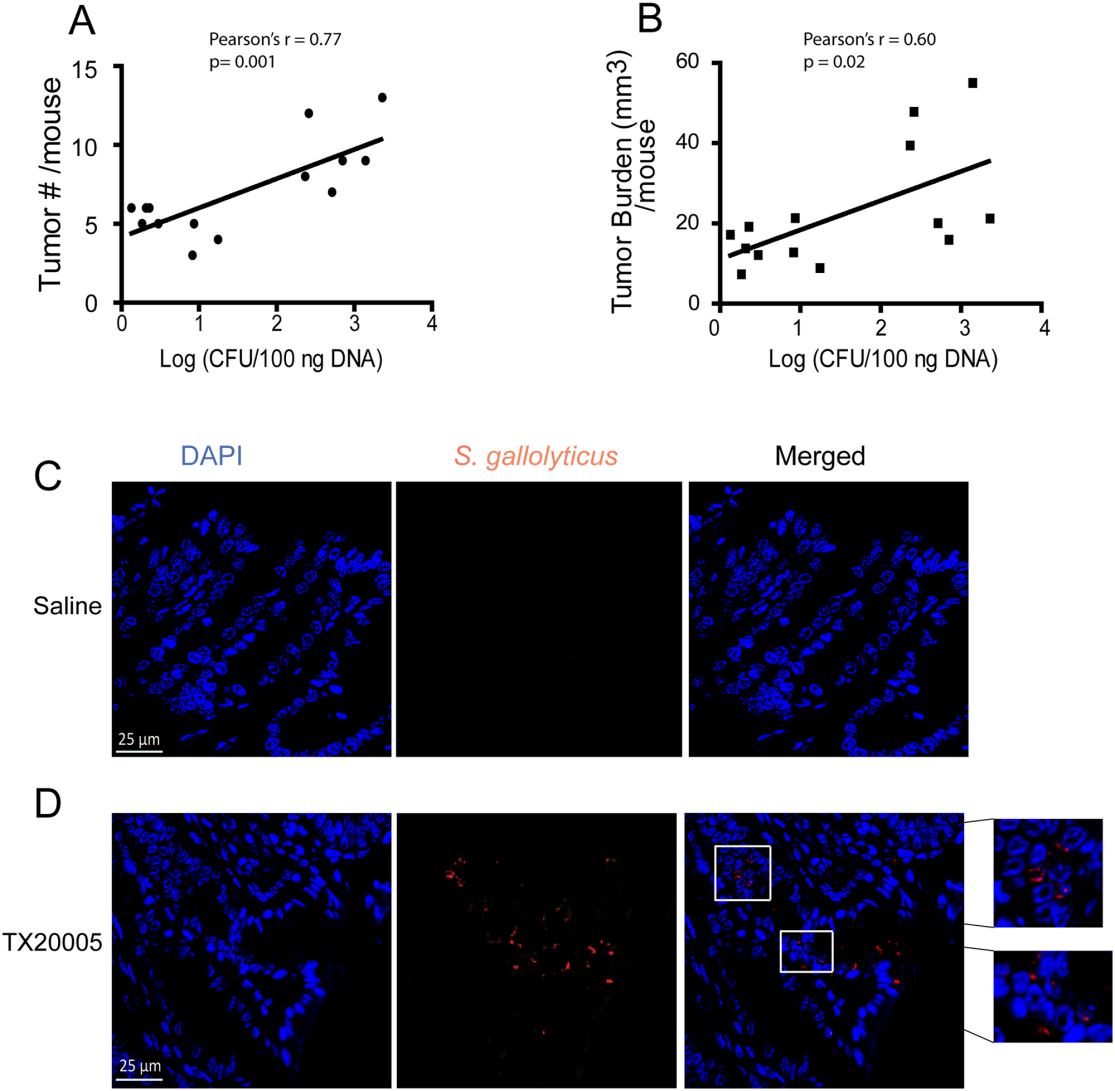
Correlation of bacterial burden with tumor number and burden. **A** and **B**. Fecal pellets were collected from mice at the end of the 12-week oral gavage of TX20005 (n = 14). DNA was extracted and analyzed by qPCR to determine the relative abundance of TX20005 as described in the Methods and Materials section. Pearson correlation analysis was performed between bacterial counts and tumor number (**A**) and burden (**B**), respectively. For tumor number, Pearson’s r = 0.77, *p* = 0.001, and for tumor burden, Pearson’s r = 0.60, *p* = 0.02. **C** and **D**. Immunofluorescence detection of *Sg* in mouse colon tumor tissues. Methcarn-fixed paraffin embedded colon sections (5 μm) from mice treated twice with AOM and 24 weeks of oral gavage with saline (**C**) or TX20005 (**D**) were incubated with anti-TX20005 antiserum and secondary antibodies as described in the Methods and Materials section. Nuclei were stained with DAPI. Scale bar represents 25μm.

To determine whether the abundance of TX20005 in the colon correlates with tumor number or burden in the mice, we collected fecal material from mice at the end of the 12-week gavage experiment. Relative abundance of *Sg* was determined by qPCR using *Sg* specific primers. We observed positive and statistically significant correlations between the relative abundance of TX20005, tumor number (Fig 8A, Pearson’s r = 0.77, *p* = 0.001), and tumor burden (Fig 8B, Pearson’s r = 0.60, *p* = 0.02), respectively. We further stained colon sections from mice treated with *Sg* or saline with anti-*Sg* antiserum. The antiserum was tested against strains of closely related species in SBSEC, as well as *Enterococcus faecalis*, *E*. *coli*, and *L*. *lactis*. The antiserum specifically recognized *Sg* but not the other strains (S15 Fig). In colon sections, we observed positive staining in *Sg*-treated mice but not in the saline control (Fig 8C and 8D), further indicating that the antiserum was specific. *Sg* bacteria were found within tumor tissues. The presence of *Sg* around normal-looking crypts was observed only occasionally, suggesting a preferential association of *Sg* with tumor tissues. Taken together, the results described above suggest that *Sg* promotes colon tumor development in the AOM-induced mouse model of CRC and this promotion involves up-regulation of β-catenin and increased colonic crypt cell proliferation.

### *S*. *gallolyticus* is present in the majority of CRC patients and preferentially associates with tumor tissues

While the association between patients with bacteremia/endocarditis caused by *Sg* and CRC is well documented, this population only represents a small proportion of CRC patients. Previous studies indicated that CRC patients might be “silently” infected with *Sg* in their colon with no symptoms of bacteremia or endocarditis [35,36]. However, the prevalence of *Sg* in CRC patients has not been extensively studied. We analyzed 148 tumors and 128 adjacent matched normal tissues from CRC patients by qPCR using *Sg*-specific primers. Overall, we found that ~74% of tumor tissues and ~47% of the normal tissues were positive for *Sg* (*p* < 0.0001, tumor vs. normal, Fisher’s exact test), suggesting that *Sg* is present in the majority of CRC patients and preferentially associates with tumor tissues. We further divided the positive samples into those with relatively high or low abundance of *Sg*. More tumor tissues were highly enriched with *Sg* (26%) than normal tissues (9%) (*p* = 0.0025, Fisher’s exact test), indicating a higher bacterial abundance in the tumor tissues (Table 1). *Streptococcus pasterianus* (*Sp*), previously *S*. *bovis* biotype II, is closely related to *Sg* however; patients with bacteremia or endocarditis due to *Sp* did not display a strong association to CRC as that reported for *Sg* in epidemiological studies [19,43,44]. The prevalence of *Sp* in CRC patients with no signs of bacteremia or endocarditis is unknown. We examined a small subset of randomly selected tumor and matched normal tissues using *Sp*-specific primers. The results showed that only 11% of tumor tissues (n = 27) and 7% of normal tissues (n = 27) were *Sp* positive, significantly lower than that of *Sg* (*p* < 0.0001, Fisher’s exact test) (Table 2). Thus the high prevalence in CRC patients is observed for *Sg* but not for a closely related organism. Taken together, these results suggest that *Sg* is present in the majority of CRC patients and preferentially associates with tumor tissues.

**Table 1:** Prevalence of *Sg* in tumor and adjacent normal tissues.

	Total	Negative	Weakly positive	Strongly positive	Total Positive
Tumor	148	38 (26%)	71 (48%)	39 (26%)	110 (74%)
Normal	128	68 (53%)	48 (38%)	12 (9%)	60 (47%)

**Table 2:** Prevalence of *Sp* in tumor and adjacent normal tissues.

	Total	Negative	Positive
Tumor	27	25 (93%)	2 (7%)
Normal	27	24 (89%)	3 (11%)

**Fig 9:**
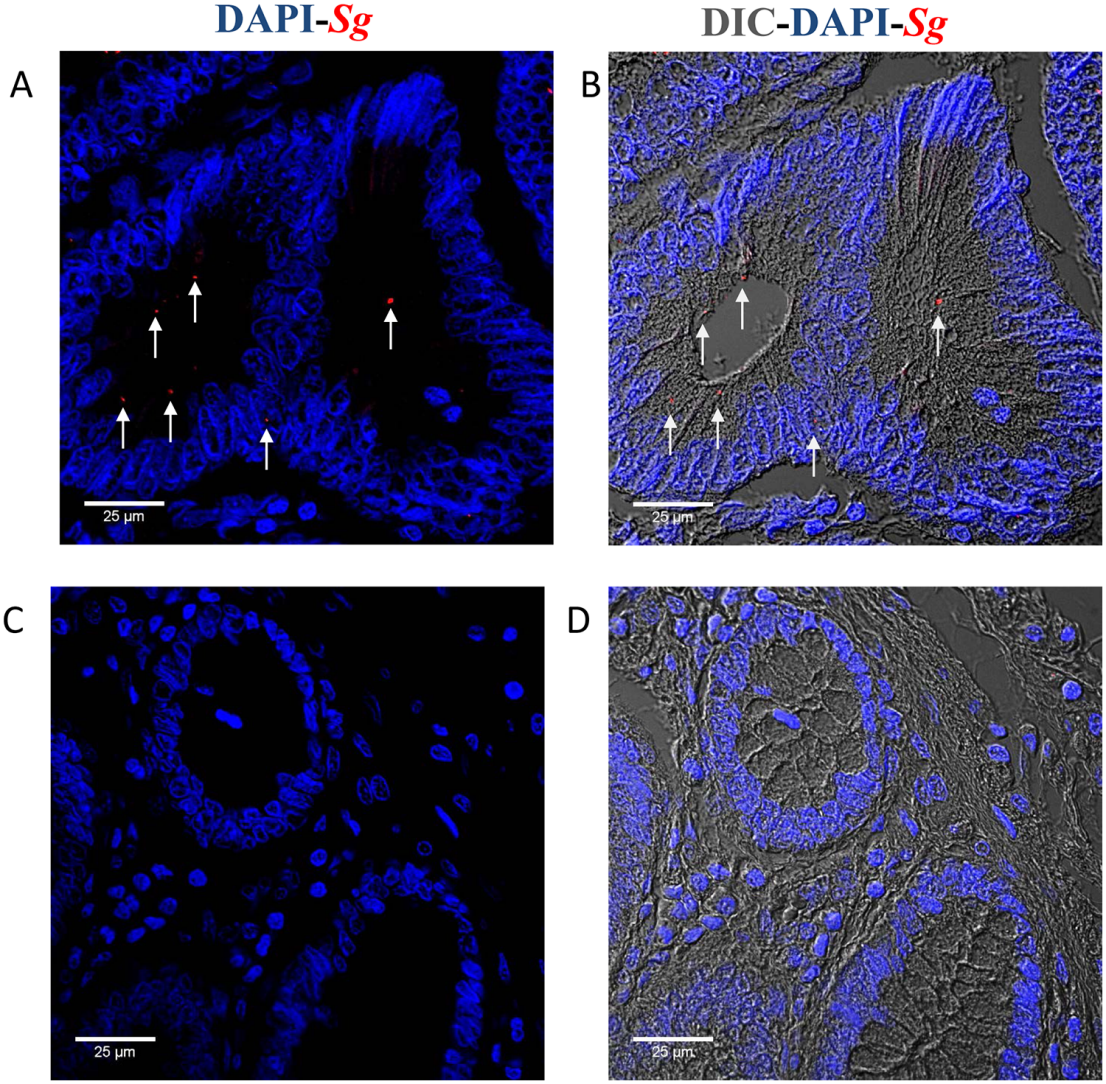
Immunofluorescence detection of *Sg* in tumor tissues from CRC patients. Formalin-fixed and paraffin embedded human tumor (stage II) (**A** and **B**) and normal tissues (**C** and **D**) were deparaffinized, rehydrated, and stained with anti-*Sg* antibodies as described in the Methods and Materials section. Nuclei were stained with DAPI. Arrows point towards *Sg*-positive staining. The scale bar represents 25μm.

Tissue sections of tumors from CRC patients were stained using *Sg*-specific antibodies to visualize the bacteria. We examined 4 normal and 21 tumor samples from CRC patients. *Sg* was detected in one of the normal samples (25%), and in 10 of the tumor samples (48%). *Sg* were seen attached to tumor tissues (Fig 9). This result is consistent with our *in vitro* observation that proximity of *Sg* to colon epithelial cells is critical for its effect on cell proliferation.

## Discussion

CRC is the second to third most common cancer and a leading cause of cancer death in the world. Annually, over a million people are diagnosed with CRC and ~700,000 die due to CRC [45]. In recent years, the role of microbial agents in the development of CRC has gained increasing recognition, raising hope that we may be able to exploit the knowledge on how microbes modulate the development of CRC to improve CRC diagnosis, prevention and treatment. To achieve this goal, a clear understanding of how precisely microbes exert their influence on tumor development is important. *Sg* infections have long been known to display a strong association with CRC, and yet virtually nothing was known about the nature of this association—*i*.*e*., whether this organism plays an active role in tumor development or its presence is merely a consequence of the tumor environment being favorable for its colonization—was unknown. The results described in this study demonstrate a tumor-promoting role of *Sg* that is dependent on cell context, specific bacterial factors, direct contact with colon cancer cells, and β-catenin signaling. We further show that *Sg* is present in the majority of CRC patients and preferentially associates with tumor tissues. Taken together, these findings highlight the importance of *Sg* in the development of CRC that extends far beyond previous recognitions.

That *Sg* promotes cell proliferation in responsive cells is based on the following results. Responsive cells co-cutlured with *Sg* had significantly higher number of viable cells, higher percentage of cells in S phase, higher level of PCNA, β-catenin (both total and nuclear), c-Myc and cyclin D1, compared to cells cultured in media only or with negative control bacteria. *Sg* did not affect the level of β-catenin or c-Myc in unresponsive cells. In addition, *Sg* did not affect cell apoptosis, as shown by a lack of sigficannt difference in annexin V staining or the level of cleaved caspase 3 between cells co-cultured with *Sg* and those with media only or with negative control bacteria.

Our results also provide evidence indicating that β-catenin is functionally important for *Sg* stimulated cell proliferation. Knockdown of β-catenin in responsive cells by two independent shRNA abolished *Sg*’s effect on cell proliferation. Treatment of cells with a β-catenin inhibitor iCRT3, which blocks the interaction between β-catenin and its partner transcriptional factor, also abolished *Sg*’s effect on cell proliferation, c-Myc and PCNA. Thus, *Sg* promotes the proliferation of responsive cells in a β-catenin dependent manner. The Wnt/β-catenin signaling pathway regulates cell proliferation and cell fate. Dysregulation of this pathway plays a central role in the development of CRC [46–50]. It is highly pertinent, therefore, that *Sg* also targets this critical pathway. The upstream events leading to the activation of β-catenin signaling upon exposure to *Sg* are unknown and are the focus of on-going studies in our laboratory. Studies on other tumor-promoting bacteria indicate that diverse strategies are used to influence β-catenin signaling. For example, *Fusobacterium nucleatum* modulates β-catenin signaling by binding to E-cadherin through its FadA adhesin [5]. *Bacteroides fragilis* secretes a zinc-dependent metalloprotease toxin that cleaves E-cadherin, leading to nuclear translocation of β-catenin, increased c-Myc expression and cell proliferation [51]. *Helicobacter pylori*, which is an important cause for gastric cancer, activates β-catenin signaling in multiple ways including affecting the expression of Wnt ligands [52], activating Wnt receptors [53], suppressing GSK3β [54,55], interfering with β-catenin/TCF4 complex by downregulating the gastric tumor suppressor *Runx3* [56–58], and interacting with E-cadherin to disrupt the E-cadherin/β-catenin complex [59]. In addition, there have been numerous studies in recent years linking microRNA (miRNA) dysregulation to CRC (recent reviews [60–64]). Evidence suggests that microbes (*e*.*g*., *H*. *pylori*, *Citrobacter rodentium*, and human papillomavirus (HPV)) can regulate β-catenin signaling and cell proliferation by affecting certain miRNAs [65–67]. However, *Sg* does not appear to encode any homologs to any bacterial factor known to modulate β-catenin or cell proliferation [68–71], suggesting the involvement of a potentially novel mechanism.

The observation that some colon cancer cell lines (SW480 and SW1116) as well as a lung cancer cell line A549 are not responsive to the effect of *Sg* suggests that *Sg* functions by engaging host factors specific to the responsive cells. All five of the colon cancer cell lines we tested contain mutations in the Wnt/β-catenin signaling pathway; HT29, LoVo, SW480 and SW1116 have mutations in APC whereas HCT116 contains a mutated version of β-catenin that results in increased protein stability [72,73]. *Sg* further increases β-catenin level in HT29, HCT116, and LoVo, but not in SW480 and SW1116 cells. It is possible that *Sg* up-regulates β-catenin at a more upstream level or by affecting factors outside the canonical Wnt/β-catenin signaling pathway. The fact that TX20005 adheres to unresponsive colon cancer cells as well as, or even better than, responsive cells suggests that the differential effects of *Sg* on responsive and unresponsive cells are not due to differences in the amount of bacteria adhering to these cells. Rather, whether or how the signal is transduced from the cell surface where *Sg* is attached is likely to be responsible for the difference. It is also possible that *Sg* adheres to different receptors on responsive and unresponsive cells. Overall, our results suggest that the effect of *Sg* depends on specific cell context. This implies that not everyone colonized by *Sg* may be equally affected; some individuals with certain genetic or epigenetic makeup may be more susceptible to the tumor-promoting effect of *Sg* than others. Identifying host factors that render cells responsive to *Sg* will be important.

The observation that bacterial species closely related to *Sg* failed to stimulate cell proliferation *in vitro* suggests the involvement of *Sg*-specific factors in this process. This finding is consistent with previous clinical observations that among the closely related species in the *S*. *bovis* group, *Sg* displays a particularly strong association with CRC. Our results also suggest that direct contact between *Sg* and responsive cells is required for *Sg*’s effect on cell proliferation. We observed that *Sg* culture supernatants or *Sg* cultured in transwell inserts were unable to stimulate cell proliferation. Furthermore, heat-killed *Sg* or *Sg* lysates had no effect on the proliferation of responsive cells. These results suggest that direct contact or close proximity of live *Sg* to host target cells is required for its stimulation of cell proliferation. We do need to point out that with respect to the requirement of live *Sg*, we cannot exclude the possibility that the relevant *Sg* factor(s) is inactivated during the heating or bacterial lysis process. The results also show that both adherence to responsive cells and promotion of cell proliferation by *Sg* depend on bacterial growth phase. Taken together, these results suggest several possibilities. Firstly, it is possible that adherence and promotion of cell proliferation is mediated by the same *Sg* surface factor(s) up-regulated in bacterial stationary phase. Secondly, it is possible that adherence brings *Sg* close to the surface of responsive cells. Subsequent actions by other *Sg* surface or secreted factors then promote cell proliferation. A third possibility is that adherence of *Sg* to responsive cells results in the up-regulation or activation of other *Sg* surface or secreted factors, which in turn stimulate cell proliferation. Studies are on-going to identify *Sg* factors involved in cell adherence and stimulation of cell proliferation. It was reported previously that an *Sg* mutant deficient of the Pil3 pilus was significantly impaired in the ability to adhere to mucus-producing cells and to colonize mouse distal colon compared to a Pil3 overexpressing *Sg* variant strain [74]. The Pil3 pilus has been shown to bind intestinal mucins and fibrinogen [75]. *Sg* also expresses a collagen binding protein Acb that mediates binding to collagen I, IV and V [70]. The genome of *Sg* encodes proteins with homology to fibrinogen/fibronectin binding proteins and major cell surface adhesion *pac*, as well as additional pilus operons [68,69]. These surface proteins can potentially play a role in *Sg* adherence to host target cells and/or colonization of the mouse colon.

The results from mouse models suggest that *Sg* promotes tumor development. *Sg*-treated responsive cells developed larger tumors in a mouse xenograft model than cells treated with control bacteria *L*. *lactis*. Higher levels of β-catenin, c-Myc and PCNA were also observed in *Sg*-treated xenografts compared to *L*. *lactis*-treated ones. In the AOM CRC model, mice treated with *Sg* had more tumors and higher tumor burden compared to *L*. *lactis* or saline-treated mice. This was confirmed using two different experimental procedures. In addition, *Sg*-treated mice had a higher percentage of proliferating cells and stronger β-catenin staining in colonic crypts compared to the control mice. Apoptosis in colon epithelial cells of *Sg*-treated mice was similar to that in *L*. *lactis*-treated mice. These findings are consistent with the results from cell culture assays. Furthermore, we observed a significant and positive correlation between *Sg* bacterial burden in the mouse colon and tumor number and burden, respectively, suggesting a dose effect. Finally, *Sg* bacteria were detected within tumor tissues from the AOM CRC model and were seen directly associated with tumor tissues from CRC patients. These results are consistent with the *in vitro* finding and suggest that *Sg* promotes tumor development by adhering to or in close proximity with tumor.

The observation that *Sg* and *L*. *lactis* induced similar levels of inflammatory responses suggests that *Sg*-induced immune responses may not play a major role in *Sg*-mediated tumor promotion. However, this does not exclude the possibility that *Sg* may induce specific types of immune reactions that favor tumor development or *Sg*’s promotion of tumor development requires a certain component of the immune system. In addition, the role of microbiota in *Sg*-mediated tumor promotion remains unclear. The results here suggest a direct effect of *Sg* on colon epithelial cells. However, whether *Sg* functions in concert with other microbial agents in the gut or elicits specific responses when mixed with certain other microbes is unknown. It is also unclear whether *Sg* plays a role in tumor initiation, since all mice were treated with AOM. Further studies are needed to clarify these issues. Overall, the results presented here support a model in which *Sg* actively promotes colon tumor growth. This promotion appears to involve up-regulation of β-catenin by *Sg*, resulting in increased cell proliferation.

Previous epidemiology studies have primarily focused on CRC risks for patients with bacteremia/endocarditis due to *Sg*. These patients only constitute a small proportion of CRC patients. Limited information was available on the prevalence of *Sg* in CRC patients with no signs of infections [35,36]. Abdulamir *et al*. studied 52 CRC patients without symptoms of bacteremia and found that approximately 33% of tumors and 23% of matched normal colon tissues were *Sg*-positive when a conventional PCR method was used for the detection of *Sg* [36]. A recent study investigated the presence of *S*. *bovis* group organisms in the colonic suction fluid from individuals who underwent colonoscopy [35]. *S*. *bovis* was isolated from each of the 17 patients diagnosed with malignant tumors. However, it was not clear if the *S*. *bovis* isolates were *Sg* or other species within the *S*. *bovis* group. Here we analyzed 148 tumor and 128 matched adjacent normal tissues from CRC patients and showed that ~ 74% of CRC patients were *Sg* positive and that *Sg* preferentially associates with tumor tissues. The difference in *Sg* prevalence between this study and others could be due to the different detection methods used, patient characteristics and/or the fact that samples were collected from different geographical regions (Malaysia [36], Israel [35], and United States (this study), respectively). Despite the difference, these studies appear to have a common theme–*Sg* is present in a much larger proportion of CRC patients than previously recognized and preferentially associates with tumor tissues. More extensive epidemiology studies are needed to further define the prevalence of *Sg* in CRC patients and its correlation with clinical, pathological and molecular characteristics of CRC. Overall, the results taken together indicate that a large proportion of CRC patients are “silently” infected with *Sg*. This information combined with our finding that *Sg* actively promotes tumor growth further highlights the clinical importance of this organism.

Previous studies have shown that *S*. *bovis* or *Sg* was isolated from fecal cultures in approximately 10% of healthy individuals [27,36]. The factors that affect *Sg* abundance in healthy individuals are unclear. It is plausible that host factors, gut microbiome composition and antibiotics usage can all have an impact. The prevalence of *Sg* in healthy individuals is lower than that in CRC patients. It was found that metabolites produced by colon cancer cells facilitate *Sg* outgrowth, resulting in a significant growth advantage of *Sg* over other bacteria in a simulated colon tumor microenvironment [76]. This could be a possible explanation for the higher prevalence of *Sg* in CRC patients. It is also possible that as tumors develop in the colon, changes in the inflammatory status and microbiome composition creates a colon environment that favors *Sg* survival and growth. Altered expression of surface proteins on tumor cells may also facilitate *Sg* adherence to the cells and colonization of the colon tissue.

In summary, this is the first report demonstrating a tumor-promoting role of *Sg*. The findings here have important clinical implications. Going forward, identifying the *Sg* factor(s) responsible for promoting cell proliferation and tumor development, and host factors targeted by *Sg*, will be critical for understanding how *Sg* functions as a tumor-promoting agent and for developing optimized strategies based on both bacterial and host characteristics to better diagnose and treat CRC.

## Methods and materials

### Bacterial strains and culture conditions

*S*. *bovis* group strains (*S*. *gallolyticus*, *S*. *pasteurianus*, *S*. *infantarius*, *S*. *macedonicus*) [70], *Lactococcus lactis* MG1363 (provided by Timothy J. Foster, Trinity College Dublin, Ireland), and *E*. *coli* XL-1 Blue were grown at 37°C in brain-heart infusion (BHI) broth with shaking or on BHI agar (Difco Laboratories, Sparks, MD). Starter cultures were prepared by growing strains overnight in 3 ml BHI broth. Fresh BHI broth was then inoculated with the overnight culture at 1:100 ratio. Cells were harvested at 0.5 OD_600nm_ for exponential phase bacteria and at 1.0 OD_600nm_ for stationary phase bacteria.

### Cell lines and growth conditions

Human colon cancer cell lines HCT116, HT29, LoVo, SW1116, SW480 and kidney epithelial cell line HEK293 (obtained from Dr. Scott Koptetz, Universoity of Texas M. D. Anderson Cancer Center, Houston, Texas and Cheryl L Walker, Baylor College of Medicine, Houston, Texas) were cultured in Dulbecco’s Modified Eagle’s Medium (DMEM, GIBCO, USA) supplemented with 10% fetal bovine serum (FBS) (GIBCO, USA). Human normal colon epithelial cell lines CCD 841 CoN and FHC were cultured in DMEM/F12 (GIBCO, USA) supplemented with 15% FBS (GIBCO, USA). Human lung carcinoma cell line A549 was maintained in F12-K media supplemented with 10% FBS. SW480, CCD 841 CoN, FHC, and A549 were purchased from ATCC. All of the cells were cultured in a humidified incubation chamber at 37°C with 5% CO_2_. HT29 stable knockdown cells were established by infecting cells with Lentiviral Transduction Particles containing short hairpin RNA (shRNA) against CTNNB1 (Sigma-Aldrich, TRCN0000314991 and TRCN0000314990) or MISSION pLKO.1-puro Non-Mammalian shRNA Control (Sigma-Aldrich, SHC002) and selected with puromycin (1μg/ml). The lentiviral *CTNNB1* shRNA plasmid or a non-mammalian shRNA control plasmid were transfected into HEK293T cells to produce lentiviral particles. Gene knockdown was confirmed by Western blot assays. All cell lines were validated based on short tandem repeats (Characterized Cell Line Core Facility, University of Texas M. D. Anderson Cancer Center, Houston, TX).

### Cell proliferation assays

Cells were seeded onto the wells of 6-well plates at 1x10^4^ cells per well and incubated for 12 hours. Exponential or stationery phase bacteria were washed with sterile phosphate buffered saline, pH 7.4 (PBS), resuspended in the appropriate cell culture media, added to the wells at 1x10^2^ cfu/well, and incubated for 24 or 48 hours. Antibiotic trimethoprim was added at 50 μg/ml final concentration after 24 hours of incubation to prevent media acidification due to bacterial growth. Trimethoprim is generally bacteriostatic for Gram-positive bacteria [77]. We have also confirmed that at the concentration we used, trimethoprim is bacteriostatic and does not kill *Sg*. Cells were detached by trypsin treatment, stained with trypan blue and counted in a Cellometer^®^ Mini automated cell counter (Nexcelome Biosciences, Lawrence, MA). Transwell experiments were performed as described above except that bacteria were cultured in Transwell inserts (Corning) with 0.4 μm pore size, which allows the passage of secreted factors and soluble metabolites but not bacteria. Each experiment was performed in duplicate and repeated at least three times. Treatment of cells with iCRT3 was based on a protocol previously described [78]. Briefly, 25 μM of iCRT3 (Sigma) was added to the cells 1 hour before the addition of bacteria. Fresh inhibitor was added every 4 hours subsequently until the end of the experiment. iCRT3 has no effect on the growth of *L*. *lactis* or *Sg*. To determine the effect of bacterial culture supernatants, stationary phase cultures grown in BHI were centrifuged and supernatants collected and filtered through a 0.2 μm filter to remove residual bacteria. 0.5 ml of a supernatant was added to each well in a 6-well culture plate.

To prepared heat killed bacteria, bacterial cells from stationary phase were incubated at 95°C for 30 mins. Bacterial lysate was prepared by using a cell disruptor (Constant Systems Ltd, UK). 100 ml of stationary phase bacterial culture was pelleted and resuspended in 10 ml PBS and then processed in the cell disruptor.

### Adherence and internalization assays

This was performed following a procedure described previously with slight modifications [79]. Cells were seeded onto the wells of 24-well tissue culture plates at 10^6^ cells/well. Bacteria from a stationary phase culture were washed twice in PBS, resuspended in DMEM supplemented with 10% FBS, and added to the wells at a multiplicity of infection (MOI) of 10. The plates were incubated in a humidified incubation chamber at 37°C with 5% CO_2_ for 1 hour. Each well was washed three times with sterile PBS to remove unbound bacteria. To determine the number of associated bacteria, cells were lysed with sterile PBS containing 0.025% Triton X-100 and dilution plated. For the detection of internalized bacteria, after washing with PBS, cells were incubated in DMEM containing 10% FBS, gentamicin (200 μg/ml) and ampicillin (200 μg/ml) for 1 hr. Cells were washed again with PBS for three times, lysed and dilution plated. All experiments were performed in triplicate wells and repeated at least three times. Adherence and internalization was expressed as a percentage of total bacteria added.

### Western blot assays

Cells were cultured in the appropriate medium in the presence or absence of bacteria for 12 hours and washed with sterile PBS three times. To obtain total cell lysates, cells were lysed with a lysis buffer (1% Triton X-100, 50 mM HEPES, pH 7.4, 150 mM NaCl, 1.5 mM MgCl_2_, 1 mM EGTA, 100 mM NaF, 10 mM Na pyrophosphate, 1 mM Na_3_VO_4_, 10% glycerol, and phosphatase inhibitor cocktail (Sigma)). To extract nuclear proteins, cells were resuspended in 200 μl of a buffer consisting of 10 mM HEPES pH 7.9, 10 mM KCl, 0.1 mM EDTA, 0.1 mM EGTA and 10 μl of 1%NP-40. Cells were vortexed for 30 sec and then centrifuged at 2000g for 5 min. Pellets were then resuspended in 100 μl of a buffer consisting of 20 mM HEPES pH 7.9, 500 mM NaCl, and 1 mM EDTA and vortexed for 10 min. Lysates were centrifuged at 14,000 rpm for 10 min and supernatants were used for western blots. To extract protein from tumor tissues, tissues were homogenized using a Tissue LyserLT (Qiagen) and lysed with the lysis buffer used for total cell lysates. The lysates were subjected to SDS-gel electrophoresis and western blot. Rabbit polyclonal antibodies against β-catenin (1:4000), c-Myc (1:3000), PCNA (1:2000), cleaved caspase 3 (1:1000), lamin B1 (1:1000) and β-actin (1:5000) were all from Cell Signaling Technology (CST). Horse radish peroxidase (HRP)-conjugated anti-rabbit IgG (CST) was used as the secondary antibody. Signals were detected using HyGLO, chemiluminescent HRP (Denville, Mteuchen, NJ). Band intensity was quantified using Image J.

### Quantitative reverse transcription PCR (RT-qPCR) to compare relative expression of cyclin D1 and cytokines

Total RNA was extracted from co-cultured cells or colon tissues using the RNeasy Kit (QIAGEN) or All-Prep DNA/RNA/Protein Mini kit (QIAGEN). cDNA was generated by using the Transcriptor First Strand cDNA Synthesis Kit (Roche). qPCR was performed using FastStart SYBR green master mix (Roche) in a Viia 7 Real Time PCR System (Applied Biosystems). The following cycle conditions were used: 95°C for 10 minutes followed by 40 cycles at 95°C for 30 seconds and 60°C for 1 minute. For cyclin D1, CT values were first normalized to GAPDH, then to cells cultured in media only (ΔΔCT). For cytokines in colon tissues, CT values were normalized β-actin (ΔCT).

### Flow cytometry

Cells were co-cultured with bacteria for 12 hours and washed with sterile PBS three times. To detect proliferating cells, cells were incubated with 5-bromodeoxyuridine (BrdU) (BD Biosciences) at a final concentration of 10 μM in cell culture media for 30 mins. Cells were washed and stained for BrdU incorporation by using BrdU Flow kit (BD Pharmingen) according to manufacturer’s instructions. For the detection of cells undergoing apoptosis, cells were stained with prodium iodide (PI) and anti-Annexin V antibodies by using Annexin V-FITC Apoptosis Detection Kit (BD Phamingen). Flow cytometry analysis of samples was done using a LSRII flow cytometer (Becton-Dickinson), and data were analyzed using the FCS Express 3 software.

### Animal experiments

All animal experiments were performed according to prototocls approved by the Institutional Animal Care and Use committee at Texas A&M Health Science Center. (1) **Xenograft model**. HCT116 or SW480 cells (1 x 10^6^) were incubated with TX20005 or *L*. *lactis* (MOI = 1) for 12 hours. The cells were immediately washed, trypsinized and mixed with Matrigel (Corning, MA) according to the manufacturer’s instructions and subcutaneously injected (100 μl) into the dorsal flap of 5-week-old nude mice (Jackson Laboratory, Bar Harbor, ME). Three hours after the injection, mice were administered a broad-spectrum antibiotic imipenem (MSD) by intraperitoneal (i.p.) injection (150 mg/kg body weight). Tumor diameters were measured with a digital caliper, and tumor volume calculated using the formula: Volume = (d1xd1xd2)/2, with d1 being the larger dimension [80]. (2) **AOM-induced mouse model of CRC**. Eight-week old female A/J mice (Jackson Laboratory, Bar Harbor, ME) were treated with AOM (10 mg/kg body weight) by i.p. injection once a week for 2 or 4 weeks. Mice were then given ampicillin (1g/L) in drinking water for one week and switched to antibiotic-free water 24 hours prior to bacterial inoculation. Mice were orally gavaged with saline, TX20005 or *L*. *lactis* using a feeding needle (~ 1 x 10^8^ cfu/mouse) at a frequency of three times per week for 24 weeks, or once a week for 12 weeks and were euthanized one week after the final gavage. One hour before sacrifice, mice received an i.p. injection of BrdU at 100 mg/kg body weight. Colons were removed by cutting from the rectal to the cecal end and opened longitudinally for visual evaluation. Tumor number was recorded and tumor size measured using a digital caliper. Tumor burden was calculated as the sum of all the tumor volumes of one mouse. Visual evaluation was carried out by two blinded observers. Mice were fed with standard ProLab IsoPro RMH3000 (LabDiet).

### Histology and immunohistochemistry

At necropsy, colons from 3 randomly selected mice from each group were “Swiss rolled” from the rectal to the cecal end, fixed in Methcarn (60% methanol, 30% chloroform, and 10% glacial acetic acid), paraffin embedded, and cut into 5μm sections across. Every 10 sections were stained with hematoxylin/eosin (H&E) and histological evaluation performed by a pathologist in a blinded fashion. Pathological scores were given using the following scale [81]: 0, no dysplasia; 1, mild dysplasia characterized by aberrant crypt foci (ACF), +0.5 for small gastrointestinal neoplasia (GIN) or multiple ACF; 2, moderate dysplasia with GIN, +0.5 for multiple occurrences or small adenoma; 3, severe or high grade dysplasia restricted to mucosa; 3.5, adenocarcinoma (involvement through muscularis mucosa); 4, adenocarcinoma (through submucosa and into or through the muscularis propria). Inflammation was scored using the following scoring matrix [82]: 0, normal; 1 - </ = 1 multifocal mononuclear cell infiltrates in lamina propria accompanied by minimal epithelial hyperplasia and slight to no depletion of mucous from goblet cells; 2, involves more of intestine or more frequent, occasional small epithelial erosions, no submucosa involvement; 3, moderate inflammation plus submucosa neutrophils, crypt abscesses, ulcers; 4, most of colon; transmural; crowding of epithelial cells with elongated crypts, ulcers plus crypt abscesses [82].

Proliferating crypt cells were detected by staining every 10 sections with anti-BrdU antibodies and counting BrdU-positive cells. A total of ~50 crypts were counted per mouse and the percentage of BrdU+ cells vs. total crypt epithelial cells counted was calculated. Apoptosis was determined by performing TUNEL assay on every 10 sections. Crypts were counted in the same manner as for BrdU+ cells. Sections were also stained for β-catenin. A Leica DM2000 LED microscope was used for imaging. Paraffin embedding, sectioning, histochemistry and immunohistochemistry were performed by the Histology Core, Gulf Coast Digestive Diseases Center, Baylor College of Medicine, Houston, TX.

### Detection of *Sg* and *Sp* by qPCR

To detect *Sg* in the mouse colon, fecal pellets were collected from mice at the end of 12-week gavage with TX20005. DNA was extracted using QIAamp Fast DNA Stool Mini Kit (Qiagen) following manufacturer’s instructions. *Sg* and *Sp*-specific primers were designed based on genomic sequences unique to *Sg* and *Sp*, respectively. The specificity of the primers was tested against a panel of strains of closely related species within the *S*. *bovis* group (S16 Fig). The primers correctly identified *Sg*, and *Sp* strains respectively, and not closely related species. The sequence for the oligonucleotides is as follows: forward *Sg*-specific primer– 5’ TGACGTACGATTGATATCATCAAC 3’, reverse *Sg*-specific primer –5’CGCTTAACACATTTTTAGCTAATACG 3’, forward *Sp*-specific primer– 5’ ATGGATAGTCATAGAATTGA 3’, and reverse *Sp*-specific primer– 5’ GGACAATGCCCTCATCTAGC 3’. qPCR was performed using Fast Plus EvaGreen qPCR Master Mix (Biotium) in a Viia 7 Real Time PCR System (Applied Biosystems) with the following cycling condition: 95°C for 10 minutes followed by 40 cycles at 95°C for 30 seconds and 60°C for 1 minute. Melting temperature analysis was performed at the end the cycles. ΔCT was normalized to the results from qPCR reactions using universal 16S rRNA primers. DNA was extracted from mouse colon tissues spiked with serially diluted *Sg* cultures of known concentration and was used to generate a standard curve. The standard curve was then used to convert ΔCT to bacterial concentration.

The same primers and cycling conditions were used to detect *Sg* and *Sp* in DNA extracted from human tumor and adjacent normal tissues. Samples enriched with *Sg* (strongly positive) is arbitrarily defined as with a 5 CT cutoff from the mean, which corresponds to approximately 90-fold enrichment above the average and approximately 750 CFU/100 ng DNA. PCR products from randomly selected positive samples were purified and sequenced. All were found to have the correct DNA sequence.

### Immunofluorescence detection of *Sg* in tumor tissues from mouse and human

Rabbit serum was raised against formalin killed TX20005 (Rockland Immunochemicals). The antiserum and pre-bleed serum were tested against TX20005, *S*. *infantarius* (TX20012), *S*. *macedonicus* (TX20026), *S*. *pasterianus* (TX20027), *E*. *coli* XL-1 Blue, and *L*. *lactis* MG 1363, to determine the specificity of the antibodies. The antiserum specifically recognized *Sg* not other bacterial strains under the experimental conditions (S15 Fig). Methcarn-fixed paraffin embedded colon sections (5 μm) from mice treated with two weekly injections of AOM and 24 weeks of oral gavage with bacteria were used to detect *Sg*. Human colon tissue sections were obtained from US Biomax. Briefly, sections were deparaffinized with xylene and rehydrated in an ethanol gradient. The slides were incubated in a citrate buffer at 95°C for 15 min, cooled to room temperature (RT), rinsed with PBS and incubated in blocking buffer (PBS containing 1% Saponin and 20% BSA) for 30 min. The slides were then incubated with rabbit anti-*Sg* serum (1:250 dilution) at 4°C overnight, washed with PBS, and incubated with donkey-anti-rabbit Alexa 594 (1:500 dilution in PBS) for 1 hr at RT. The slides were washed again, stained with DAPI, mounted and examined in a DeltaVision Elite microscope (GE Healthcare).

### Statistical analyses

Comparisons of multiple groups were done by two-tailed one-way or two-way analysis of variance (ANOVA) followed by Student-Newman-Keuls (SNK) test. Comparison between two groups of data was done by unpaired, two-tailed t test. Pearson correlation analysis was performed to determine the correlation between *Sg* burden and tumor number and burden, respectively. Fisher’s exact test was used to compare the qPCR data from human tissues. Analyses were carried out using the Graphpad Prism 6 software.

### Ethics statement

Animal studies were performed in accordance with protocols (IACUC#2014-0370-IBT) approved by the Institutional Animal Care and Use Committee at the Texas A&M Health Science Center, Institute of Biosciences and Technology. The Texas A&M University Health Science Center—Institute of Biosciences and Technology is registered with the Office of Laboratory Animal Welfare per Assurance A4012-01. It is guided by the PHS Policy on Human Care and Use of Laboratory Animals (Policy), as well as all applicable provisions of the Animal Welfare Act.

Colon biopsy samples were collected from patients at the University of Texas M. D. Anderson Cancer Center (MDACC), Houston, TX. The patients had previously given written informed consent for use of their samples in future colorectal cancer research. Patient identifiers were anonymized. The mean age at surgery is 62.5. The cohort contains mostly stage II and III tumors. Collection and handling of patient samples were carried out in strict accordance to protocols approved by the institutional review board at MDACC and Texas A&M Health Science Center.

## Supporting information

S1 Fig Flow cytometry analysis of the effect of *Sg* on colon cancer cell proliferation. Cells (~1x10^5^ /well) were incubated with *L*. *lactis*, TX20005 or media only for 12 hrs. Cells were then pulsed with 10 mM BrdU for 30 min. BrdU incorporation was determined by flow cytometry, as described in the Methods and Materials section. **A**. HCT116. **B**. HT29. **C**. FHC. The experiment was repeated 3 times. Representative histograms are shown.
(PDF)

S2 Fig Flow cytometry analysis of the effect of *Sg* on apoptosis. Cells (~1x10^5^/well) were incubated with *L*.*lactis*, TX20005 or media only for 12 hrs. Cells were then stained with anti-Annexin V antibodies and then incubated with propidium iodide (PI). The percentage of apoptotic cells is indicated. **A**. HCT116. **B**. HT29. **C**. FHC. The experiment was repeated 3 times. Representative histograms are shown.
(PDF)

S3 Fig Detection of cleaved caspase 3 in cells treated with *Sg*. Approximately 1x10^5^ cells/well were incubated with media only, *L*. *lactis* or TX20005 (~1x10^5^ cfu/well) for 12 hrs in a 6 well plate. Whole cell lysates were prepared as described in the Methods and Materials section and analyzed by Western blot assays. **A**. HCT116; **B**. HT29; **C**. FHC. The experiment was repeated three times and representative images are shown.
(PDF)

S4 Fig Promotion of cell proliferation requires *Sg*-specific factors. **A** and **B**. Species closely related to *Sg* do not promote cell proliferation. Stationary phase bacteria were added to HT29 (**A**) and HCT116 (**B**) cells, co-cultured for 48 hours and viable cell numbers enumerated. TX20005, TX20030 and TX20031, *S*. *gallolyticus* (*Sg)*; TX20012, *S*. *infantarius (Si)*; TX20026, *S*. *macedonicus (Sm)*; TX20027, *S*. *pasteurianus (Sp)*. Data are presented as the mean ± SEM. Data analyzed by unpaired, two-tailed Student’s t tests. Each experiment was done with duplicate wells and was repeated at least three times. *, *p* < 0.05; **, *p* < 0.01.
(PDF)

S5 Fig Bacterial growth under co-culture conditions. Approximately 1x10^4^ cells/well were incubated with *L*. *lactis*, TX20005, TX20026, TX20027 or TX20012 (~1 x 10^2^ cfu/well) for 48 hrs in a 6 well plate. Samples were collected at indicated time points and diluation plated to determine bacterial counts. The experiment was repeated twice and results were presented as the mean ± SEM. Two-way two-tailed ANOVA was performed to compare the growth curves of the different strains. There was no significant difference between the different strains.
(PDF)

S6 Fig *Sg* culture supernatant had no effect on cell proliferation. HT29 (**A**) and HCT116 (**B**) cells were co-cultured with bacterial supernatant or bacterial cells collected from stationary phase TX20005 culture. Cells were then incubated for 24 hours as in cell proliferation assays described in the Methods and Materials section. Cell numbers are normalized to the untreated samples at 24 hours. Each experiment was done with duplicate wells and was repeated at least three times. Data are presented as the mean ± SEM. Statistical analysis was performed using unpaired, two-tailed t tests. *, *p* < 0.05.
(PDF)

S7 Fig Heat killed *Sg* or *Sg* lysates do not promote cell proliferation in responsive colon cancer cells. HT29 cells (~ 1x10^4^ cells/well) were incubated with 100 μl of heat killed *Sg* or bacterial lysates prepared by sonication, as described in the Methods and Materials section. After 24 hours of incubation, cells were detached by trypsin treatment, stained with trypan blue and counted in an automated cell counter. Each experiment was done with duplicate wells and was repeated at least three times. Cell numbers are normalized to cells incubated with media only at 24 hours. Data is presented as the mean ± SEM. Data was analyzed by two-tailed one-way ANOVA followed by SNK test. *, *p* < 0.05.
(PDF)

S8 Fig Adherence to and internalization of *Sg* by the host cells. Adherence and internalization of *Sg* strains TX20005 and TX20030 to different cell lines was performed as described in the Methods and Materials section. Briefly, stationary or exponential phase bacteria were incubated with indicated host cells for 1 hour. Cells were washed, lysed and dilution plated to determine the amount of total attached bacteria. For internalization, after washing cells were incubated in media containing gentamicin, washed, lysed and dilution plated. Adherence and internalization was expressed as the percentage of adhered or internalized bacteria vs. total bacteria added. **A**. Adherence of stationary TX20005 and TX20030 to various cell lines. **B**. Internalization of stationary TX20005 and TX20030 by various cell lines. **C**. Adherence of stationary and exponential phase TX20005 and TX20030 to HT29 cells. All experiments were performed in triplicate wells and repeated at least three times. Data are presented as the mean ± SEM. Statistical analysis was performed using unpaired, two-tailed t tests. *, *p* < 0.05;**, *p* < 0.01; ***, *p* < 0.001.
(PDF)

S9 Fig Sg did not increase the level of β-catenin or c-Myc in A549 cells. Approximately 1x10^5^ A549 cells/well were incubated with media only, *L*. *lactis* or TX20005 (~1 x 10^5^ cfu/well) for 12 hrs in a 6 well plate. Whole cell lysates were prepared as described in the Methods and Materials section and analyzed by western blot assays. The experiment was repeated two times. Representative images are shown.
(PDF)

S10 Fig **A**. β-catenin level in untransfected HT29 colon cancer cells (lanes 1–2), HT29 cells transfected with control shRNA (lanes 3–4) and HT29 cells transfected with β-catenin specific shRNAs (lanes 5–6) as assessed by immunoblotting using total cell lysates. **B**. Knockdown of β-catenin abolished the effect of Sg on cell proliferation. β-catenin stable knockdown HT29 cells (HT29-B2) or HT29 cells transfected with a control shRNA (HT29-C2) were seeded into the wells of 6-well plates at ~1x10^4^ cells/well and incubated for 12 hours. Stationary phase bacteria were added to the wells at ~1x10^2^ cfu/well, and incubated for 24 or 48 hours. Cells were stained with trypan blue and viable cells counted in an automated cell counter. Data in panel **B** was analyzed by two-way two-tailed ANOVA followed by SNK test. Data in panel **C** was analyzed one-way two-tailed ANOVA followed by SNK test. Data are presented as the mean ± SEM. Each experiment was done with duplicate wells and was repeated at least three times. *, *p* < 0.05.
(PDF)

S11 Fig Xenograft experiment using unresponsive SW480 cells. ~ 1 x 10^6^ SW480 cells were treated with TX20005 or *L*. *lactis*, mixed with Matrigel and injected into the dorsal flap of nude mice (n = 5/group) as described in the Methods and Materials section. Tumor size was measured during the indicated time period with a digital caliper. Cells co-cultured with TX20005 were compared with cells co-cultured with *L*. *lactis*. Data are presented as the mean ± SEM. Data was analyzed by unpaired, two-tailed t tests.
(PDF)

S12 Fig TUNEL assay of colon sections from mice treated with AOM and bacteria. A/J mice were administered with 2 weekly i.p. injections of AOM, followed by treatment with Amp (1g/L) in drinking water for 1 week and oral gavage of saline, *L*.*lactis*, or TX20005 for 24 weeks. Methcarn-fixed colon sections (5 μm) were subject to TUNEL assays to detect apoptotic cells. (n = 3/group). Scale bar represents 50 μm.
(PDF)

S13 Fig Cytokine levels in tumor and normal colon tissues from mice gavaged with *Sg* or *L*. *lactis*. At necropsy, tumor and adjacent normal tissues were collected from mice in the two AOM treatment group and immediately stored in liquid nitrogen. RNA extraction and RT-qPCR was performed as described in the Methods and Material section. ΔCT was normalized to the results from the qPCR reactions using β-actin primers. N, normal colon tissue; T, tumor tissue.
(PDF)

S14 Fig *Sg* promotes colon tumor development in an AOM-induced mouse model of CRC. A/J mice were administered with 4 weekly i.p. injections of AOM, followed by treatment with Amp (1g/L) in drinking water for 1 week and oral gavage of *L*. *lactis* (n = 17), TX20005 (n = 19) or saline (n = 17) for 12 weeks. Colons were visually examined to for macroscopic tumors. Tumor size was measured and tumor burden was calculated as described in the Methods and Materials section. Data are presented as the mean ± SEM. Data was analyzed by unpaired, two-tailed t tests. *, *p* < 0.05.
(PDF)

S15 Fig Specificity of anti-*Sg* antibodies serum. Bacteria were attached to poly-L-lysine coated coverslips, fixed with 2% paraformaldehyde, blocked with PBS containing 5% goat serum, and incubated with rabbit anti-TX20005 serum (1:250) or pre-bleed serum (1:250), followed by donkey anti-rabbit Alexa Fluor (1:1000).
(PDF)

S16 Fig Specificity of *Sg* and *Sp* primers. PCR was carried out using bacterial cells as template as described in the Methods and Material section. From left to right, DNA ladder, *Sg* strains ATCC BAA-2069, TX20005, TX20030, TX20031, TX20034, *Sm* strain TX20026, *Sp* strain TX20027, and *Si* strain TX20012.
(PDF)
