# Supplementary figures and images for: Streptococcus gallolyticus subsp. gallolyticus promotes colorectal tumor development
Source: PLoS Pathog. 2017 Jul 13;13(7):e1006440. doi: 10.1371/journal.ppat.1006440 (PMC5509344; doi:10.1371/journal.ppat.1006440)

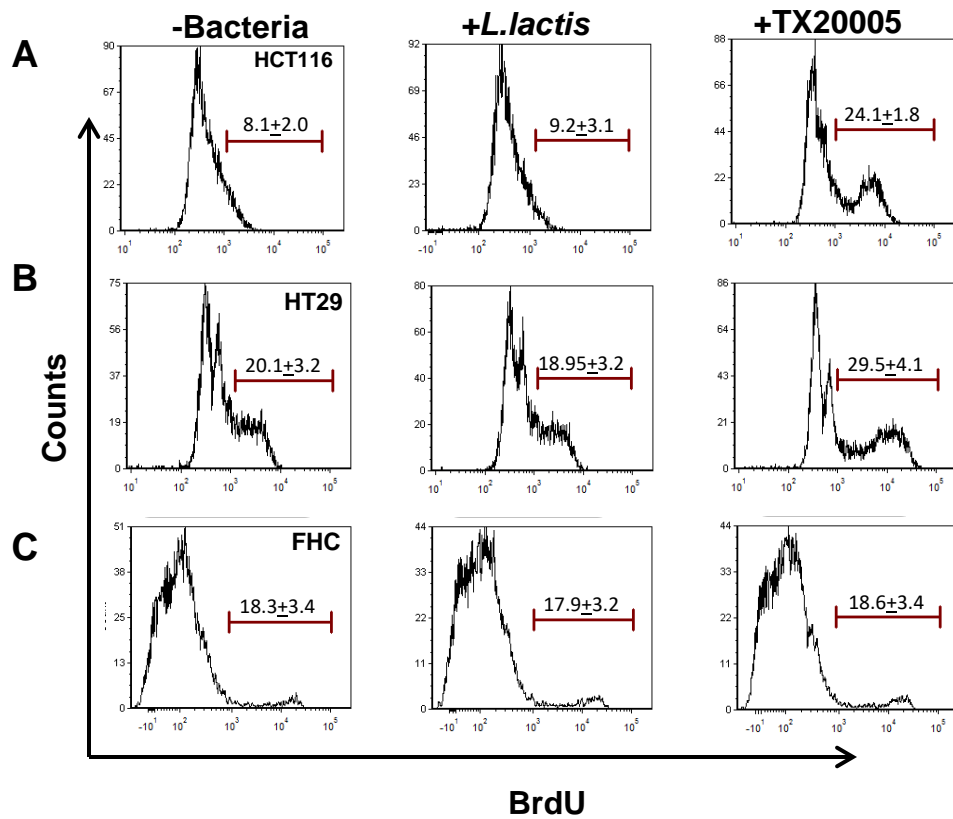

**S1 Fig**

Supplement: S1 Fig — Cells (~1x10^5^ /well) were incubated with L. lactis, TX20005 or media only for 12 hrs. Cells were then pulsed with 10 mM BrdU for 30 min. BrdU incorporation was determined by flow cytometry, as described in the Methods and Materials section. A. HCT116. B. HT29. C. FHC. The experiment was repeated 3 times. Representative histograms are shown. (PDF) [file ppat.1006440.s001.pdf]

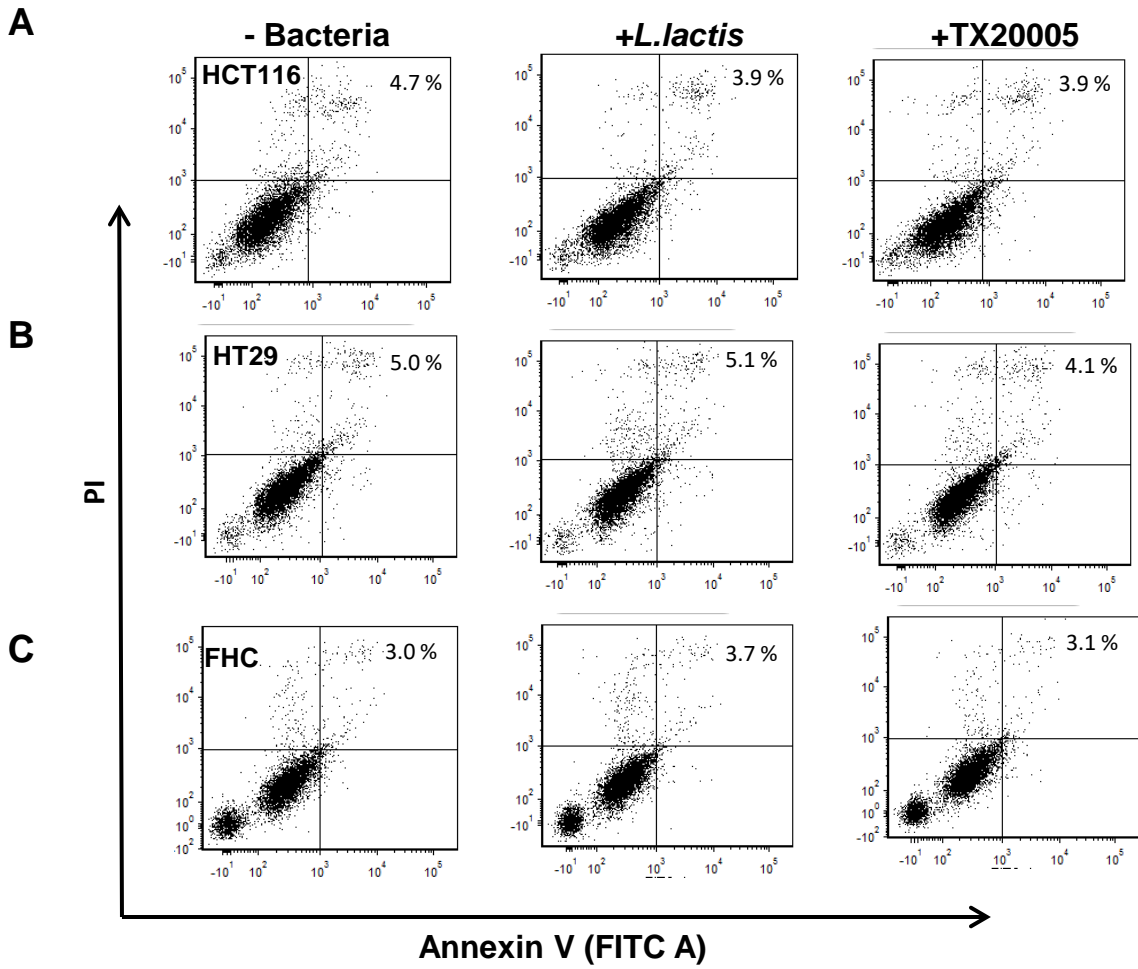

**S2 Fig**

Supplement: S2 Fig — Cells (~1x10^5^/well) were incubated with L.lactis, TX20005 or media only for 12 hrs. Cells were then stained with anti-Annexin V antibodies and then incubated with propidium iodide (PI). The percentage of apoptotic cells is indicated. A. HCT116. B. HT29. C. FHC. The experiment was repeated 3 times. Representative histograms are shown. (PDF) [file ppat.1006440.s002.pdf]

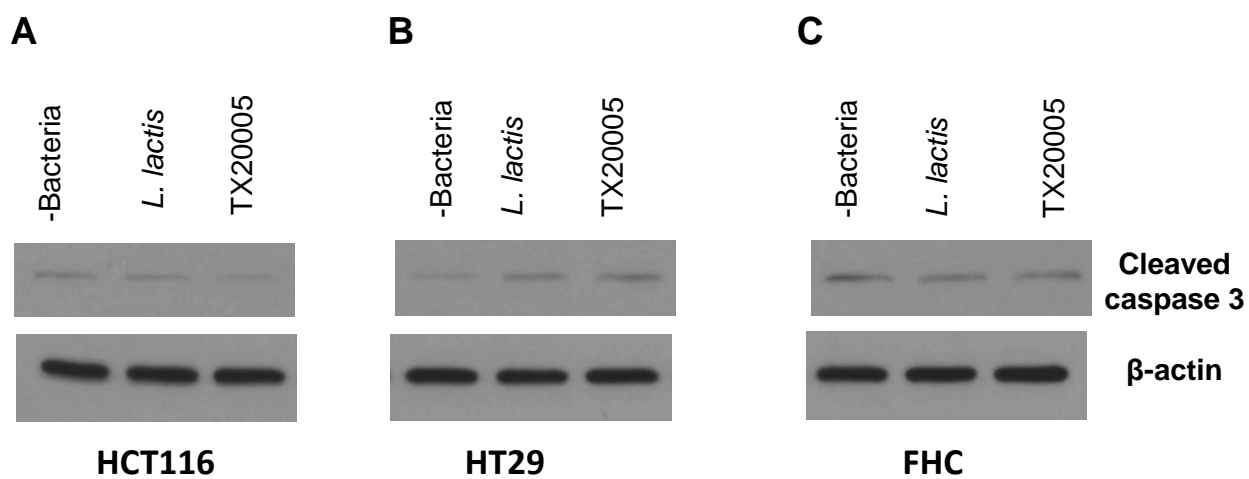

**S3 Fig**

Supplement: S3 Fig — Approximately 1x10^5^ cells/well were incubated with media only, L. lactis or TX20005 (~1x10^5^ cfu/well) for 12 hrs in a 6 well plate. Whole cell lysates were prepared as described in the Methods and Materials section and analyzed by Western blot assays. A. HCT116; B. HT29; C. FHC. The experiment was repeated three times and representative images are shown. (PDF) [file ppat.1006440.s003.pdf]

**A**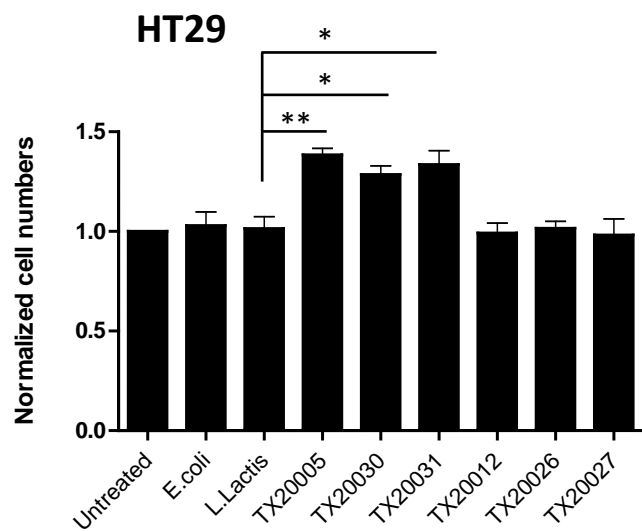**B**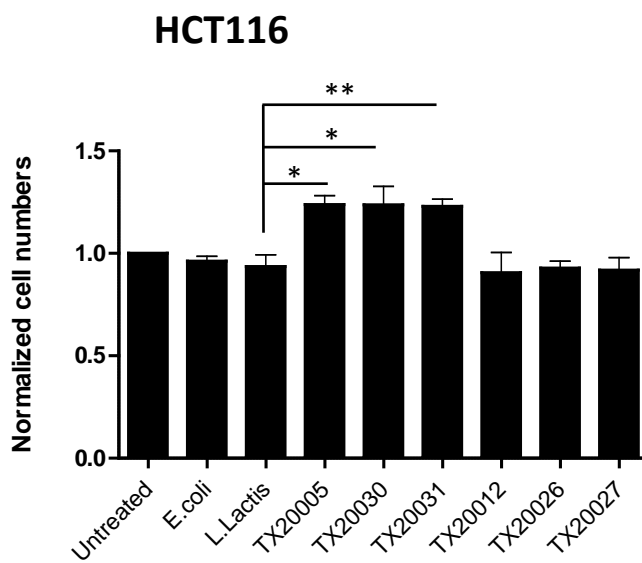**S4 Fig**

Supplement: S4 Fig — A and B. Species closely related to Sg do not promote cell proliferation. Stationary phase bacteria were added to HT29 (A) and HCT116 (B) cells, co-cultured for 48 hours and viable cell numbers enumerated. TX20005, TX20030 and TX20031, S. gallolyticus (Sg); TX20012, S. infantarius (Si); TX20026, S. macedonicus (Sm); TX20027, S. pasteurianus (Sp). Data are presented as the mean ± SEM. Data analyzed by unpaired, two-tailed Student’s t tests. Each experiment was done with duplicate wells and was repeated at least three times. *, p < 0.05; **, p < 0.01. (PDF) [file ppat.1006440.s004.pdf]

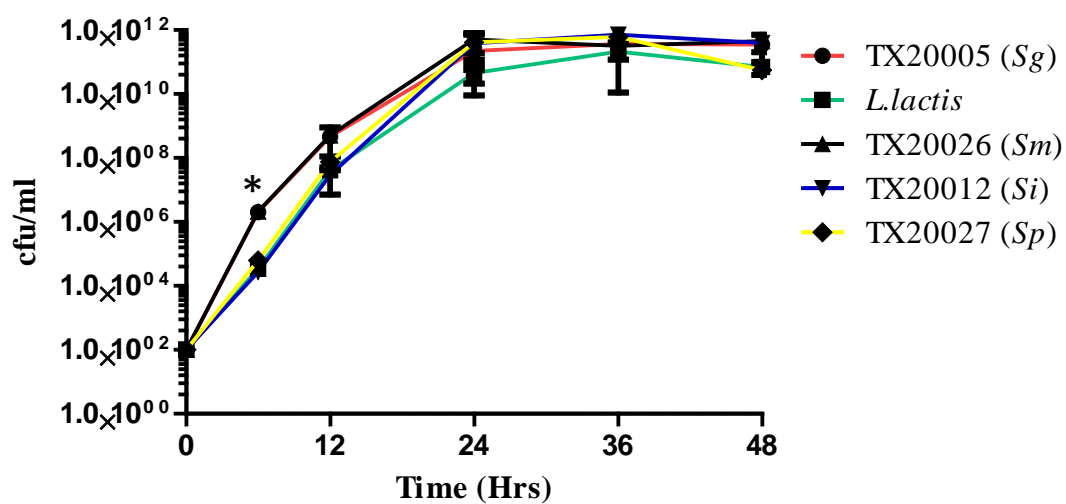

S5 Fig

Supplement: S5 Fig — Approximately 1x10^4^ cells/well were incubated with L. lactis, TX20005, TX20026, TX20027 or TX20012 (~1 x 10^2^ cfu/well) for 48 hrs in a 6 well plate. Samples were collected at indicated time points and diluation plated to determine bacterial counts. The experiment was repeated twice and results were presented as the mean ± SEM. Two-way two-tailed ANOVA was performed to compare the growth curves of the different strains. There was no significant difference between the different strains. (PDF) [file ppat.1006440.s005.pdf]

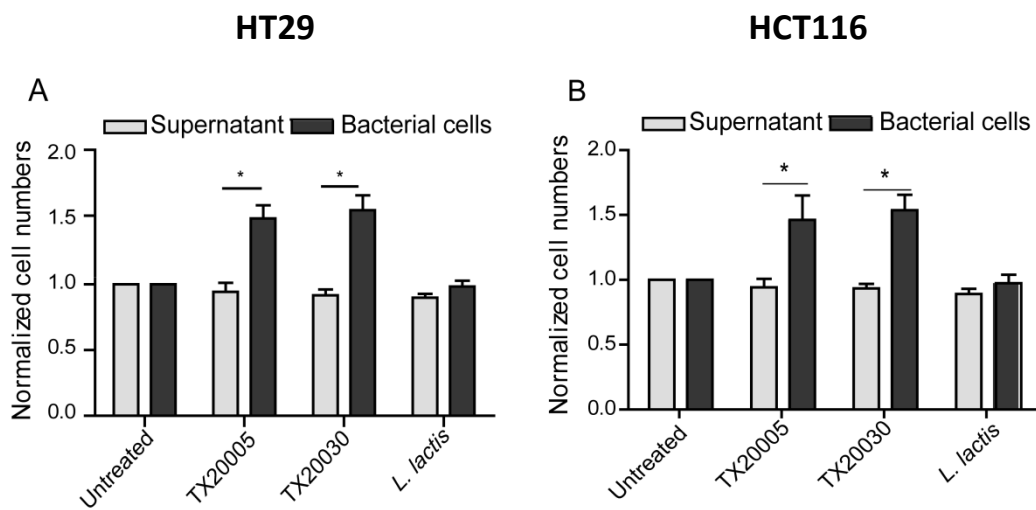

**S6 Fig**

Supplement: S6 Fig — HT29 (A) and HCT116 (B) cells were co-cultured with bacterial supernatant or bacterial cells collected from stationary phase TX20005 culture. Cells were then incubated for 24 hours as in cell proliferation assays described in the Methods and Materials section. Cell numbers are normalized to the untreated samples at 24 hours. Each experiment was done with duplicate wells and was repeated at least three times. Data are presented as the mean ± SEM. Statistical analysis was performed using unpaired, two-tailed t tests. *, p < 0.05. (PDF) [file ppat.1006440.s006.pdf]

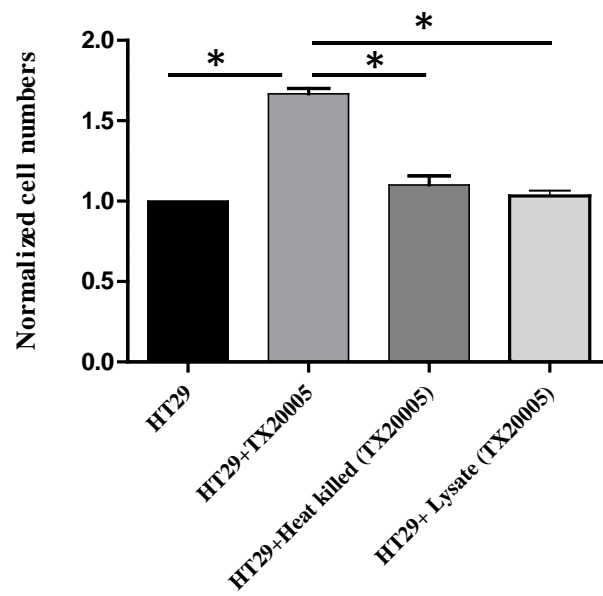

**S7 Fig**

Supplement: S7 Fig — HT29 cells (~ 1x10^4^ cells/well) were incubated with 100 μl of heat killed Sg or bacterial lysates prepared by sonication, as described in the Methods and Materials section. After 24 hours of incubation, cells were detached by trypsin treatment, stained with trypan blue and counted in an automated cell counter. Each experiment was done with duplicate wells and was repeated at least three times. Cell numbers are normalized to cells incubated with media only at 24 hours. Data is presented as the mean ± SEM. Data was analyzed by two-tailed one-way ANOVA followed by SNK test. *, p < 0.05. (PDF) [file ppat.1006440.s007.pdf]

**A**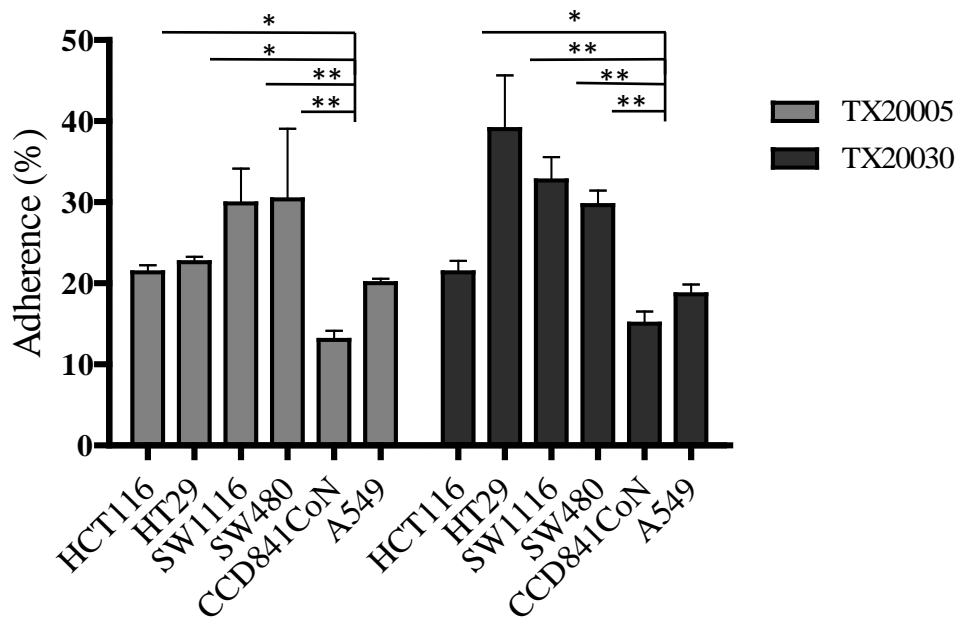**B**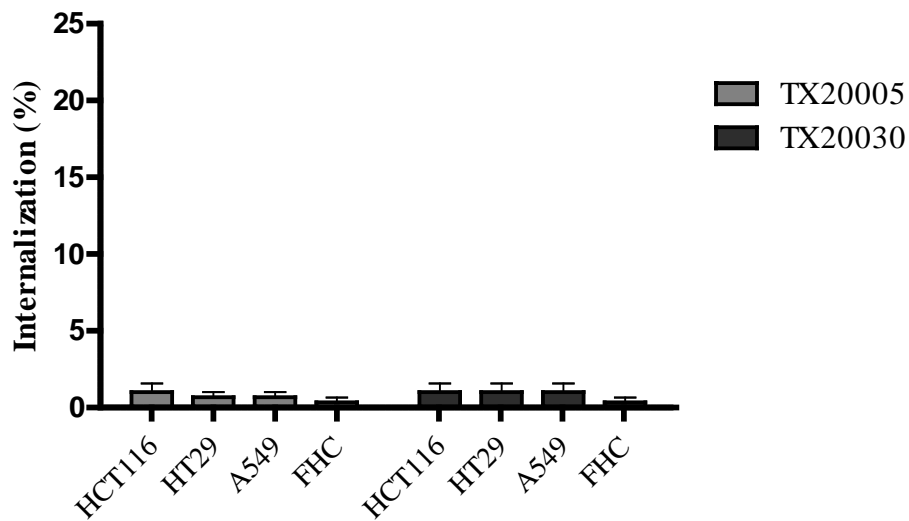**C**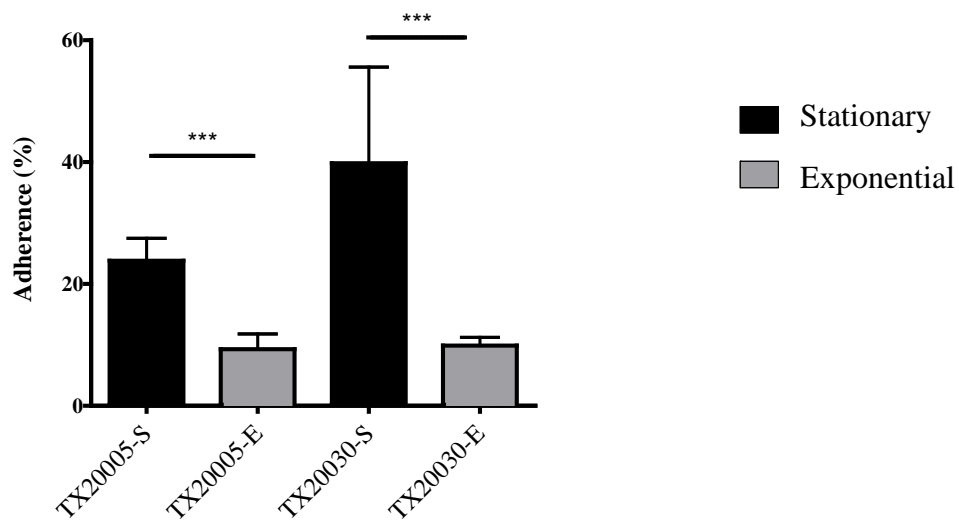**S8 Fig**

Supplement: S8 Fig — Adherence and internalization of Sg strains TX20005 and TX20030 to different cell lines was performed as described in the Methods and Materials section. Briefly, stationary or exponential phase bacteria were incubated with indicated host cells for 1 hour. Cells were washed, lysed and dilution plated to determine the amount of total attached bacteria. For internalization, after washing cells were incubated in media containing gentamicin, washed, lysed and dilution plated. Adherence and internalization was expressed as the percentage of adhered or internalized bacteria vs. total bacteria added. A. Adherence of stationary TX20005 and TX20030 to various cell lines. B. Internalization of stationary TX20005 and TX20030 by various cell lines. C. Adherence of stationary and exponential phase TX20005 and TX20030 to HT29 cells. All experiments were performed in triplicate wells and repeated at least three times. Data are presented as the mean ± SEM. Statistical analysis was performed using unpaired, two-tailed t tests. *, p < 0.05;**, p < 0.01; ***, p < 0.001. (PDF) [file ppat.1006440.s008.pdf]

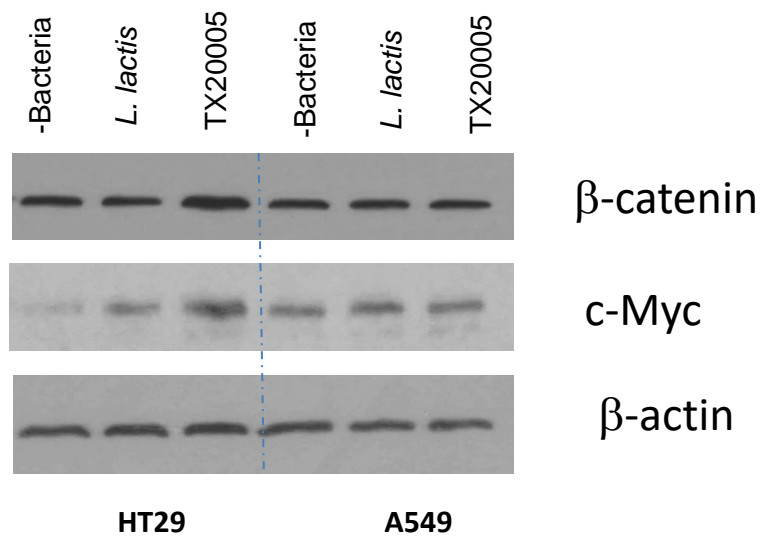

**S9 Fig**

Supplement: S9 Fig — Approximately 1x10^5^ A549 cells/well were incubated with media only, L. lactis or TX20005 (~1 x 10^5^ cfu/well) for 12 hrs in a 6 well plate. Whole cell lysates were prepared as described in the Methods and Materials section and analyzed by western blot assays. The experiment was repeated two times. Representative images are shown. (PDF) [file ppat.1006440.s009.pdf]

**A**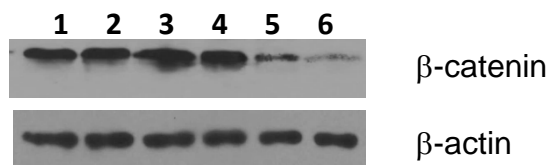**B**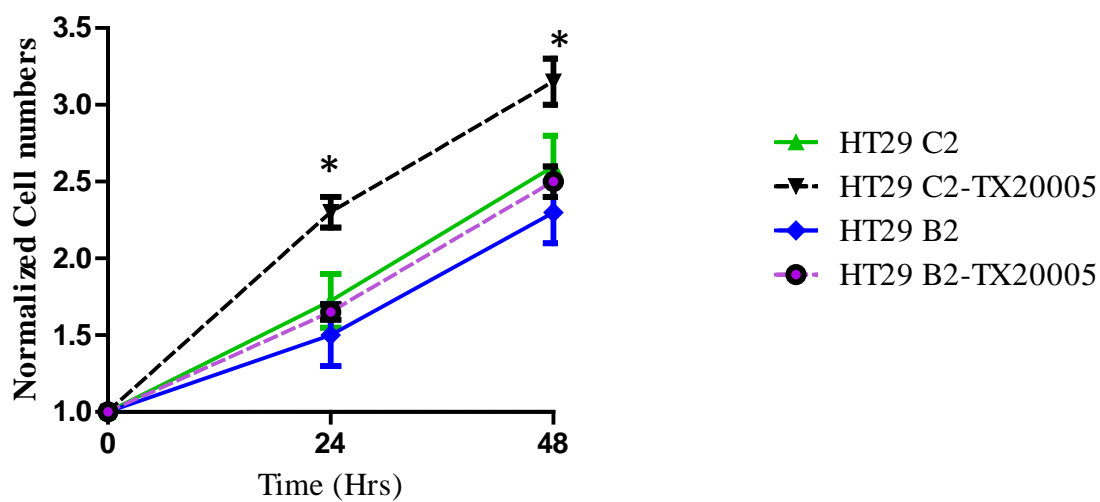**C**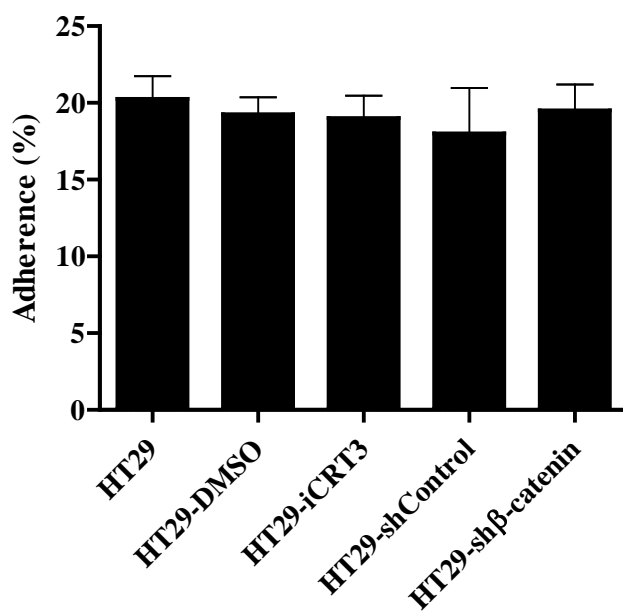**S10 Fig**

Supplement: S10 Fig — A. β-catenin level in untransfected HT29 colon cancer cells (lanes 1–2), HT29 cells transfected with control shRNA (lanes 3–4) and HT29 cells transfected with β-catenin specific shRNAs (lanes 5–6) as assessed by immunoblotting using total cell lysates. B. Knockdown of β-catenin abolished the effect of Sg on cell proliferation. β-catenin stable knockdown HT29 cells (HT29-B2) or HT29 cells transfected with a control shRNA (HT29-C2) were seeded into the wells of 6-well plates at ~1x10^4^ cells/well and incubated for 12 hours. Stationary phase bacteria were added to the wells at ~1x10^2^ cfu/well, and incubated for 24 or 48 hours. Cells were stained with trypan blue and viable cells counted in an automated cell counter. Data in panel B was analyzed by two-way two-tailed ANOVA followed by SNK test. Data in panel C was analyzed one-way two-tailed ANOVA followed by SNK test. Data are presented as the mean ± SEM. Each experiment was done with duplicate wells and was repeated at least three times. *, p < 0.05. (PDF) [file ppat.1006440.s010.pdf]

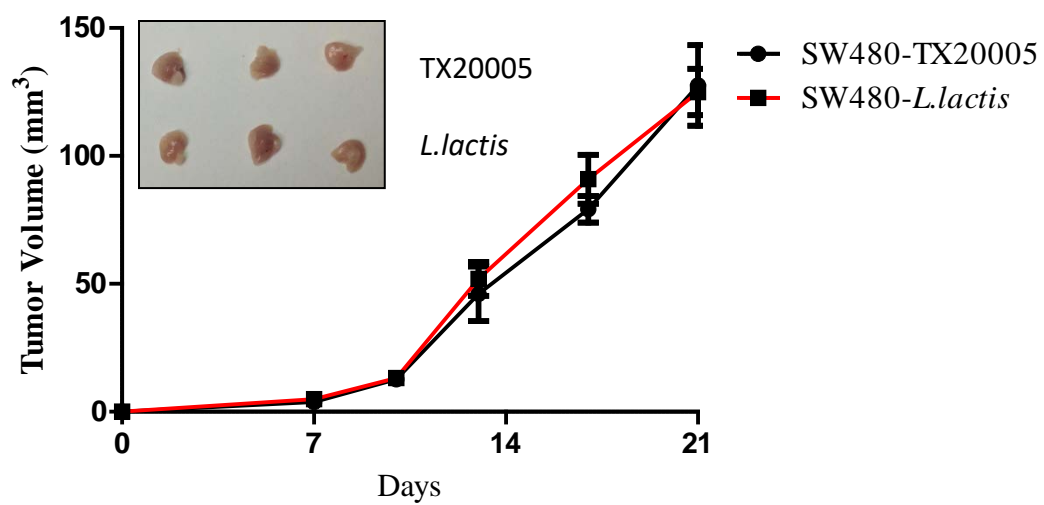

**S11 Fig**

Supplement: S11 Fig — ~ 1 x 10^6^ SW480 cells were treated with TX20005 or L. lactis, mixed with Matrigel and injected into the dorsal flap of nude mice (n = 5/group) as described in the Methods and Materials section. Tumor size was measured during the indicated time period with a digital caliper. Cells co-cultured with TX20005 were compared with cells co-cultured with L. lactis. Data are presented as the mean ± SEM. Data was analyzed by unpaired, two-tailed t tests. (PDF) [file ppat.1006440.s011.pdf]

**Saline**

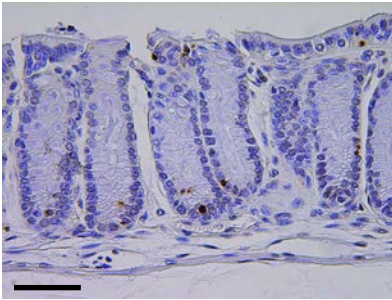

***L. lactis***

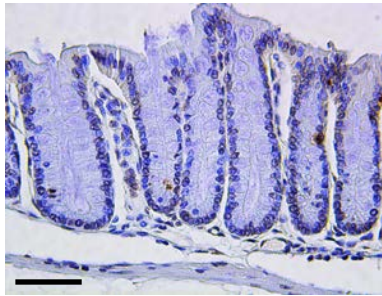

**TX20005**

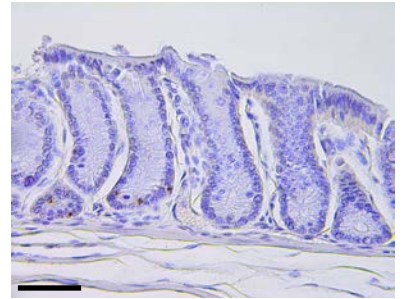

**S12 Fig**

Supplement: S12 Fig — A/J mice were administered with 2 weekly i.p. injections of AOM, followed by treatment with Amp (1g/L) in drinking water for 1 week and oral gavage of saline, L.lactis, or TX20005 for 24 weeks. Methcarn-fixed colon sections (5 μm) were subject to TUNEL assays to detect apoptotic cells. (n = 3/group). Scale bar represents 50 μm. (PDF) [file ppat.1006440.s012.pdf]

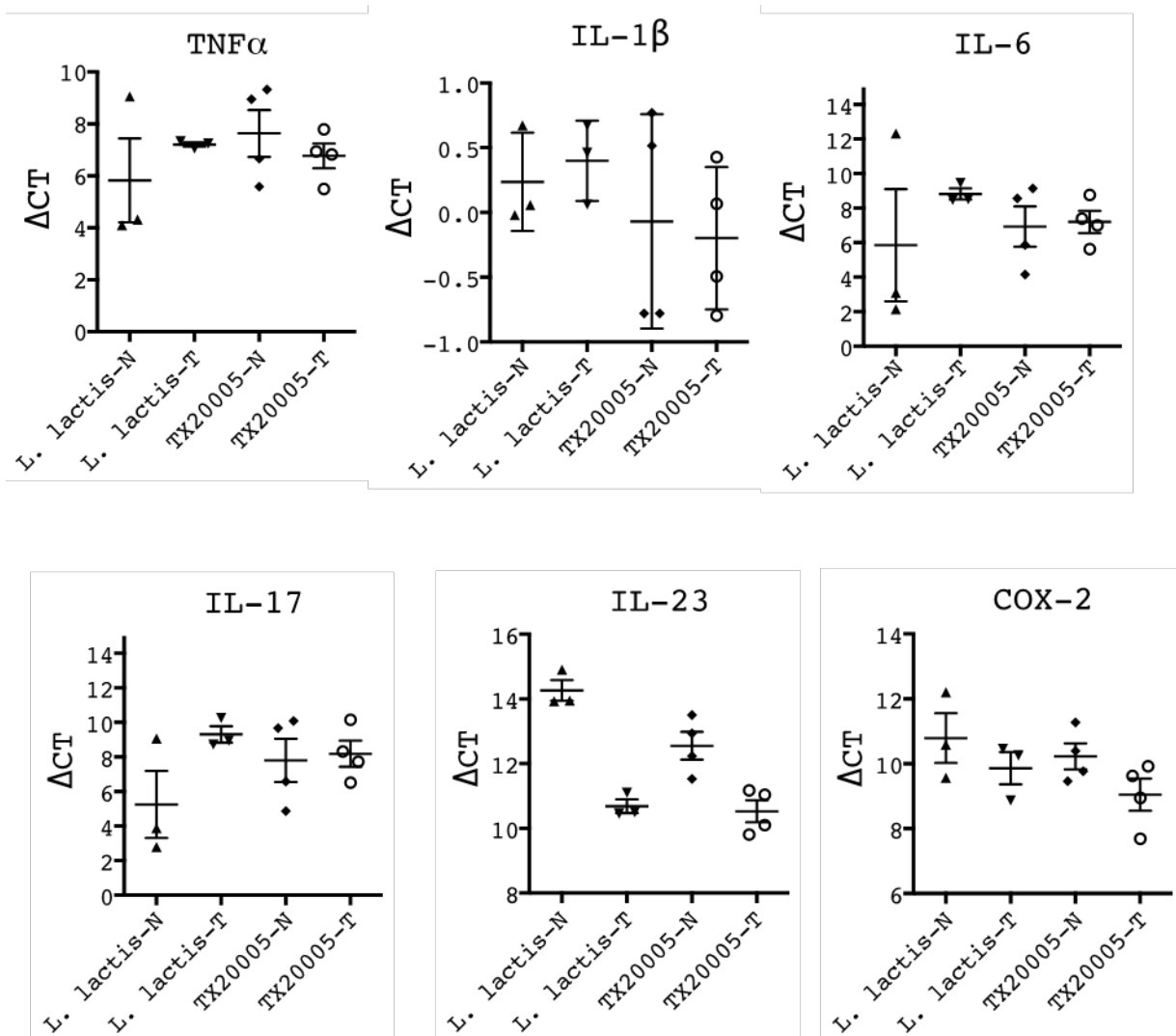

**S13 Fig**

Supplement: S13 Fig — At necropsy, tumor and adjacent normal tissues were collected from mice in the two AOM treatment group and immediately stored in liquid nitrogen. RNA extraction and RT-qPCR was performed as described in the Methods and Material section. ΔCT was normalized to the results from the qPCR reactions using β-actin primers. N, normal colon tissue; T, tumor tissue. (PDF) [file ppat.1006440.s013.pdf]

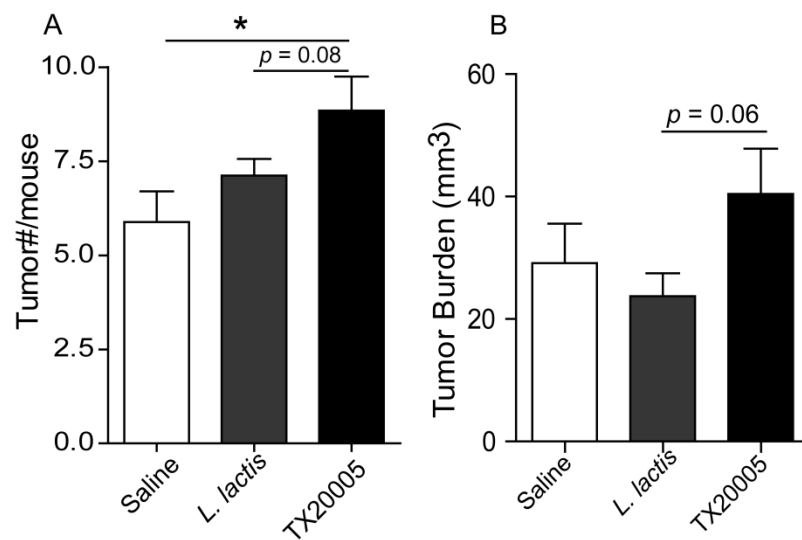

**S14 Fig**

Supplement: S14 Fig — A/J mice were administered with 4 weekly i.p. injections of AOM, followed by treatment with Amp (1g/L) in drinking water for 1 week and oral gavage of L. lactis (n = 17), TX20005 (n = 19) or saline (n = 17) for 12 weeks. Colons were visually examined to for macroscopic tumors. Tumor size was measured and tumor burden was calculated as described in the Methods and Materials section. Data are presented as the mean ± SEM. Data was analyzed by unpaired, two-tailed t tests. *, p < 0.05. (PDF) [file ppat.1006440.s014.pdf]

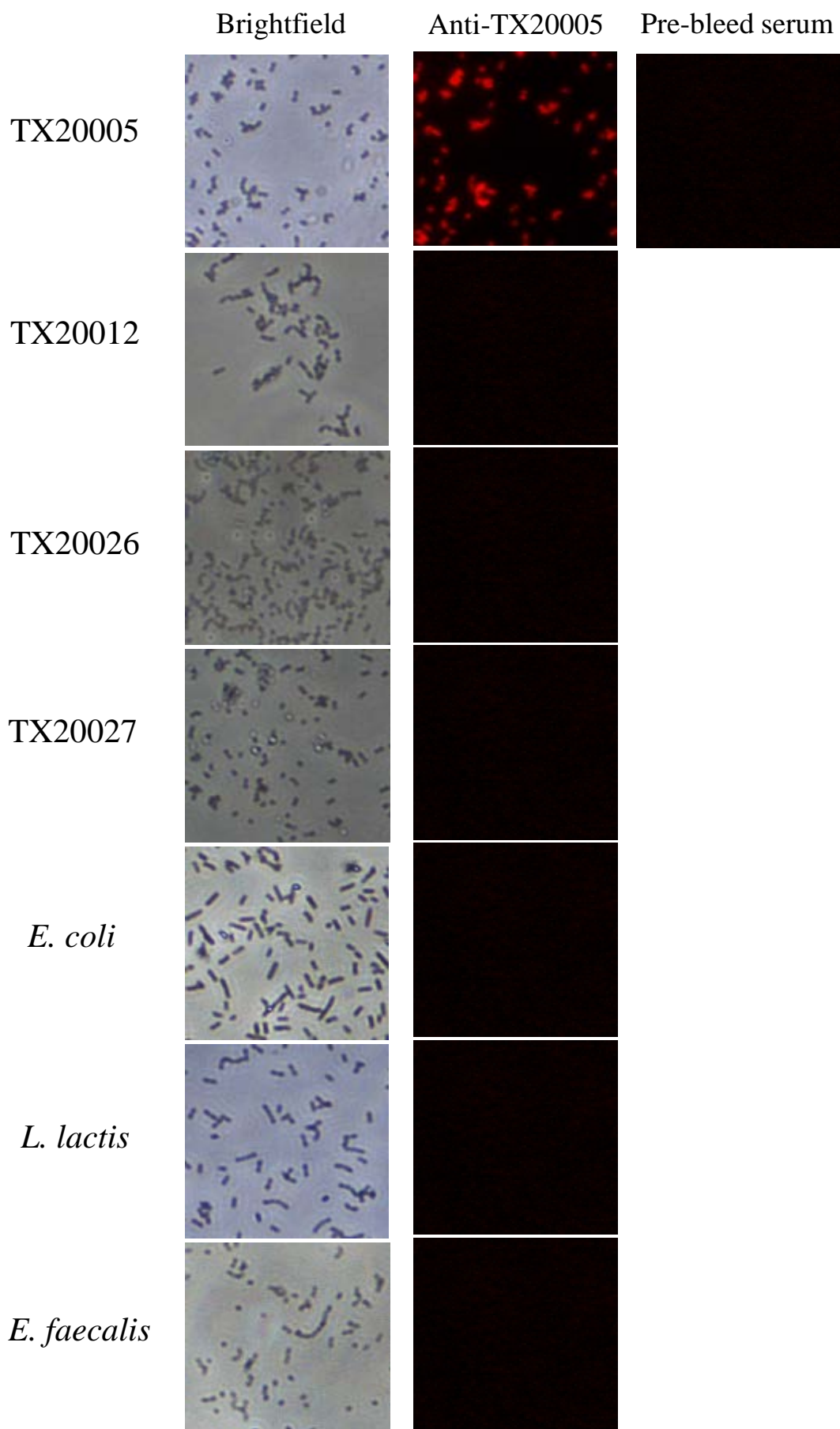

S15 Fig

Supplement: S15 Fig — Bacteria were attached to poly-L-lysine coated coverslips, fixed with 2% paraformaldehyde, blocked with PBS containing 5% goat serum, and incubated with rabbit anti-TX20005 serum (1:250) or pre-bleed serum (1:250), followed by donkey anti-rabbit Alexa Fluor (1:1000). (PDF) [file ppat.1006440.s015.pdf]

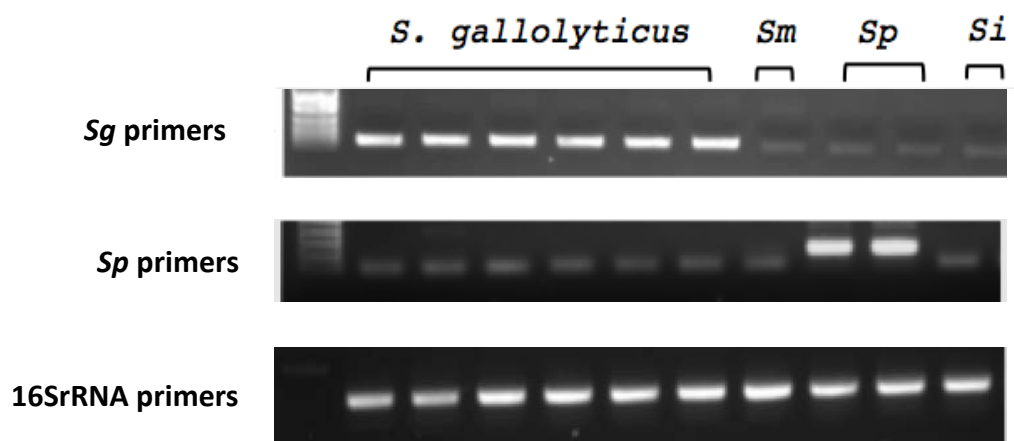

**S16 Fig**

Supplement: S16 Fig — PCR was carried out using bacterial cells as template as described in the Methods and Material section. From left to right, DNA ladder, Sg strains ATCC BAA-2069, TX20005, TX20030, TX20031, TX20034, Sm strain TX20026, Sp strain TX20027, and Si strain TX20012. (PDF) [file ppat.1006440.s016.pdf]
